# Maternal plasma levels of oxytocin during breastfeeding—A systematic review

**DOI:** 10.1371/journal.pone.0235806

**Published:** 2020-08-05

**Authors:** Kerstin Uvnäs­Moberg, Anette Ekström-Bergström, Sarah Buckley, Claudia Massarotti, Zada Pajalic, Karolina Luegmair, Alicia Kotlowska, Luise Lengler, Ibone Olza, Susanne Grylka-Baeschlin, Patricia Leahy-Warren, Eleni Hadjigeorgiu, Stella Villarmea, Anna Dencker

**Affiliations:** 1 Department of Animal Environment and Health, Swedish University of Agricultural Sciences, Skara, Sweden; 2 Department of Health Sciences, University of West, Trollhättan, Sweden; 3 School of Public Health, The University of Queensland, Herston, QLD, Australia; 4 Academic Unit of Obstetrics and Gynecology, University of Genova, Genova, Italy; 5 Physiopathology of Human Reproduction Unit, IRCCS Ospedale Policlinico San Martino, Genova, Italy; 6 Faculty of Health Studies, Campus Diakonhjemmet, VID Specialized University, Oslo, Norway; 7 Berufs Bildung Zentrum Gesundheit Ingolstadt, Ingolstadt, Germany; 8 Department of Clinical & Experimental Endocrinology, Faculty of Health Sciences with Subfaculty of Nursing and Institute of Maritime and Tropical Medicine, Medical University of Gdańsk, Gdańsk, Poland; 9 Midwifery Education Unit, Freiburg University Medical Center, Freiburg, Germany; 10 Faculty of Medicine, University of Alcalá, Alcalá de Henares, Spain; 11 Research Unit for Midwifery Science, Zurich University of Applied Sciences, Winterthur, Switzerland; 12 School of Nursing and Midwifery, University College Cork, Cork, Ireland; 13 Nursing Department, Health Science, Cyprus University of Technology, Limassol, Cyprus; 14 Faculty of Philosophy, University of Alcalá, Alcalá de Henares, Spain; 15 Faculty of Philosophy, University of Oxford, Oxford, United Kingdom; 16 Institute of Health and Care Sciences, Sahlgrenska Academy, University of Gothenburg, Göteborg, Sweden; TNO, NETHERLANDS

## Abstract

**Introduction:**

Oxytocin is a key hormone in breastfeeding. No recent review on plasma levels of oxytocin in response to breastfeeding is available.

**Materials and methods:**

Systematic literature searches on breastfeeding induced oxytocin levels were conducted 2017 and 2019 in PubMed, Scopus, CINAHL, and PsycINFO. Data on oxytocin linked effects and effects of medical interventions were included if available.

**Results:**

We found 29 articles that met the inclusion criteria. All studies had an exploratory design and included 601 women. Data were extracted from the articles and summarised in tables. Breastfeeding induced an immediate and short lasting (20 minutes) release of oxytocin. The release was pulsatile early postpartum (5 pulses/10 minutes) and coalesced into a more protracted rise as lactation proceeded. Oxytocin levels were higher in multiparous versus primiparous women. The number of oxytocin pulses during early breastfeeding was associated with greater milk yield and longer duration of lactation and was reduced by stress. Breastfeeding-induced oxytocin release was associated with elevated prolactin levels; lowered ACTH and cortisol (stress hormones) and somatostatin (a gastrointestinal hormone) levels; enhanced sociability; and reduced anxiety, suggesting that oxytocin induces physiological and psychological adaptations in the mother. Mechanical breast pumping, but not bottle-feeding was associated with oxytocin and prolactin release and decreased stress levels. Emergency caesarean section reduced oxytocin and prolactin release in response to breastfeeding and also maternal mental adaptations. Epidural analgesia reduced prolactin and mental adaptation, whereas infusions of synthetic oxytocin increased prolactin and mental adaptation. Oxytocin infusion also restored negative effects induced by caesarean section and epidural analgesia.

**Conclusions:**

Oxytocin is released in response to breastfeeding to cause milk ejection, and to induce physiological changes to promote milk production and psychological adaptations to facilitate motherhood. Stress and medical interventions during birth may influence these effects and thereby adversely affect the initiation of breastfeeding.

## Introduction

The European Cooperation in Science and Technology (EU COST), supports scientific collaboration within the European Union. This article was created within the framework of the COST Action IS1405 BIRTH: "Building Intrapartum Research Through Health—An interdisciplinary whole system approach to understanding and contextualising physiological labour and birth" (http://www.cost.eu/COST_Actions/isch/IS1405). A literature review article regarding plasma levels of oxytocin in connection with labour and birth, performed within this COST action has already been published [1].

Breastfeeding is recognized as the best way to feed infants and the World Health Organization (WHO) recommends exclusive breastfeeding for the first six months of life [2, 3]. Breastfeeding gives rise to many positive effects in babies and their mothers. It for example protects against infections in new-borns and early in life and it may also reduce the occurrence of some allergic conditions, obesity, and type 1 diabetes later on in childhood. In addition, it has also been suggested to increase the level of intelligence [4]. For mothers, breastfeeding stimulates physiological and psychological changes in order to facilitate milk production and adaptations to motherhood. In addition, it gives long-term protection against diseases such as breast cancer, ovarian cancer, cardiovascular disease and diabetes type 2 [4, 5].

Breastfeeding also supports the interaction and bonding between mother and infant. Longer duration of breastfeeding was associated with more maternal sensitive responsiveness and more attachment security and less attachment disorganization in the child [6].

Oxytocin, a peptide molecule produced in the supraoptic and paraventricular nuclei (SON and PVN) of the hypothalamus, plays a key role during breastfeeding. Oxytocin released into the circulation during breastfeeding promotes milk ejection by contracting the myoepithelial cells surrounding the mammary gland alveoli and by relaxing the milk duct sphincters.

Suckling also induces a release of oxytocin from nerves within the brain where oxytocin facilitates both physiological and psychological adaptations for breastfeeding and motherhood. Oxytocin promotes prolactin release and thereby milk production. It also induces powerful anti-stress effects, including decreased blood pressure and cortisol levels, and stimulates digestive and metabolic processes [7].

The release of oxytocin into the circulation and the brain occurs in parallel in response to each breastfeeding episode. A recent publication provides an elaborate description of the mechanisms by which the release of oxytocin into the circulation and brain are coordinated [1].

It has been suggested that medical interventions during labour and birth, including caesarean section [8], epidural analgesia [9], and infusion of synthetic oxytocin [10] might negatively impact the initiation and/or continuation of breastfeeding. Medical interventions may hypothetically cause such effects by influencing the release of and effects caused by oxytocin during labour and birth in the long term.

The aim of this study was to review plasma levels of oxytocin levels in response to breastfeeding. Also, other oxytocin linked effects presented in the identified articles as well as data regarding effects of medical interventions during labour and birth are reported. Data obtained within the same article allows more detailed investigations of the relationship between oxytocin and oxytocin linked effects, than does data presented in separate articles. The same applies for articles on the effects of medical interventions.

## Methods

A systematic literature search, with the aim to summarize the knowledge regarding oxytocin levels in response to breastfeeding, was carried out according to the PRISMA statement [11] and using Covidence©, an online platform for systematic reviews [12]. The main inclusion criterion was plasma levels of oxytocin in response to breastfeeding, All the *inclusion criteria* are shown in Table 1.

**Table 1:** Inclusion criteria.

**Inclusion criteria**
**Population: **Breastfeeding women with and without medical interventions during labour, birth, or immediately postpartum.
The breastfeeding women should have had at least two plasma oxytocin measurements in connection to breastfeeding or breast pumping
**Outcome: **Maternal oxytocin levels in blood/plasma
**Included articles: **Peer- reviewed original research/data
All types of study designs using any technique of oxytocin analysis
No limitation in years
**Languages: **English, German, French, Japanese

The search strings were created by KUM, AEB and SB together with librarians from the University of Queensland and searches were performed in the following databases: Pubmed, Scopus, CINAHL, and PsycINFO in September 2017. An additional literature search based on the same search string was performed in September 2019.

### Procedure for selection of studies

In 2017, 453 articles were identified via database searches (PubMed n = 305, Scopus n = 91, CINAHL n = 7, and PsycINFO n = 50). Fifteen additional articles were identified through other sources. Forty duplicates were removed from the 468 articles leaving 428 articles. By screening of titles and abstracts, 268 of the 468 articles were excluded. After full-text screening of the remaining 160 articles, 28 articles were identified that met the inclusion criteria, were included in this review. A last search was performed in September 2019. Additional 34 papers were identified. After removing duplicates and screening of titles and abstracts, two full text papers were included and reviewed. One paper was excluded due to no reporting of oxytocin levels [13] and one paper met the inclusion/exclusion criteria [14].

All together 29 articles were identified in which maternal oxytocin levels were recorded during breastfeeding or other types of breast stimulation [14–42]. Note that the 29 included articles are based on 25 clinical studies, as data from 3 of the clinical studies had been split and published in 2 separate articles, thus [20, 34] and [30, 32] and [36, 37] were based on the same clinical studies. For 2 of the other included articles, Jonas et. al. 2009 [24] and Uvnäs Moberg et. al. 1990 [37] additional material regarding breastfeeding induced effects had been published as separate articles [43–45] and [46, 47] respectively. Data from these additional studies have also been included in the tables. Altogether 601 women participated in the studies summarized.

**Fig 1:**
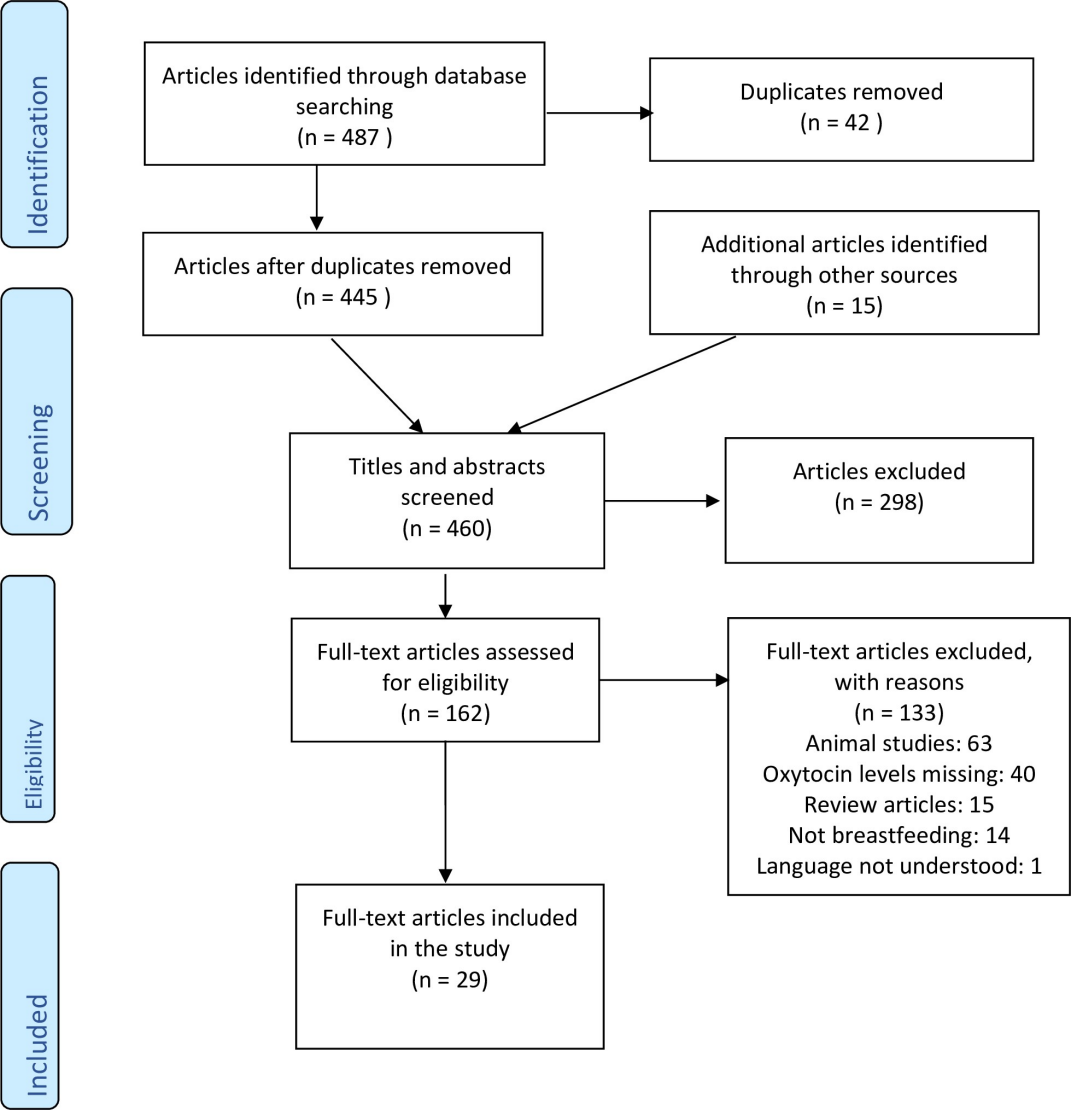
Flow diagram of study selection.

At each stage, articles were screened by at least two authors working independently in pairs (AEB, KUM, CM, KL, SB & ZP) based on the inclusion and exclusion criteria (Table 1). In case inclusion was unclear, an expert third author (KUM) was involved. Final decisions were agreed by SB & KUM. The selection process is illustrated in a flow diagram (Fig 1).

### Procedure for data extraction and compilation of data

The articles included in this study are mainly of an explorative research design and are not randomized, controlled, intervention studies. Data were extracted from the articles and compiled into tables. KUM, SB, AEB, AK, ZP, LL and KL participated in this work. Characteristics of the included articles are presented in Table 2, data on oxytocin levels in Tables 3 and 4, prolactin levels in Table 5, variables related to stress in Table 6, variables related to digestion and nutrition in Table 7 and variables related to personality and mood in Table 8. If data on associations between oxytocin and the secondary effects of oxytocin was available, these data were also included in the tables. Data on effects of medical interventions on breastfeeding induced oxytocin levels are presented separately (Table 4).

**Table 2:** Characteristics of the included clinical studies.

Author, year and title	Number of participating women *(Breastfeeding unless otherwise stated)*	Timing of experiment	Data of importance for interpretation of the results	Recorded variables and other publications based on the included research studies
**I: Amico & Finlay, 1986 [15]** Breast stimulation in cycling women, pregnant women and a woman with induced lactation: pattern of release of oxytocin, prolactin and luteinizing hormone	19 pregnant	Third trimester	Breast stimulation with mechanical pump or tactile stimulation	Oxytocin
	5 normal cycling	Luteal phase	Mechanical pump, tactile or sham stimulation (rubbing chest wall)	Prolactin
	1 with induced lactation	5, 40, 90 days post-adoption	Breast stimulation or suckling	Uterine activity (pregnant women only)
**II: Amico et. al., 1994 [16]** Suckling-induced attenuation of plasma cortisol concentrations in postpartum lactating women	6	Various times between 1–24 weeks postpartum	Suckling	Oxytocin
				Cortisol
**III: Chatterton et. al., 2000 [17]** Relation of plasma oxytocin and prolactin concentrations to milk production in mothers of preterm infants: influence of stress	18	7, 14, 21, 28, 35 and 42 days postpartum	All infants preterm (<30 weeks) and not suckling at the breast Breast pumping with single or double mechanical pump	Oxytocin
				Prolactin
				Cortisol (saliva)
				Alpha-amylase (saliva)
				Milk volume per week
**IV: Chiodera et al., 1991 [18]** Relationship between plasma profiles of oxytocin and adrenocorticotropic hormone during suckling or breast stimulation in women	7	5–7 days postpartum	Suckling	Oxytocin
			Same women were exposed to sham stimulation (rubbing chest wall) 2 days later as control	ACTH
	6 normal fertile women	Day 21 and 23 of menstrual cycle	Breast stimulation with mechanical pump or sham stimulation 2 days later as control	Cortisol
**V: Christensson et.al., 1989 [19]** Effect of nipple stimulation on uterine activity and on plasma levels of oxytocin in full term, healthy, pregnant women	10 pregnant	38–39 weeks of gestation	Manual nipple stimulation	Oxytocin
				Uterine activity
				Fetal heart rate
**VI a: Cox et. al., 2015 [20]** Oxytocin and HPA stress axis reactivity in postpartum women	39	2–8 weeks postpartum	Suckling	Oxytocin
	8 non-breastfeeding		Holding or formula feeding	Prolactin
				CRF
**VI b: Stuebe et. al., 2013 [34]** Association between maternal mood and oxytocin response to breastfeeding				Cortisol
				Oestradiol
				Progesterone
				T4
				Heart rate
(VI a and VI b; articles based on the same material)				Stress reactivity
				EPDS (depression)
				STAI-State and STAI-Trait (anxiety)
**VII: Dawood, et.al., 1981 [21]** Oxytocin release and plasma anterior pituitary and gonadal hormones in women during lactation	12	3–5 days postpartum	Suckling	Oxytocin
				Prolactin
				Thyroid stimulating hormone
				Follicle stimulating hormone
				Luteinising hormone
				Oestrone
				Oestradiol
				Progesterone
**VIII: Erickson et. al., (2019) [14].** Oxytocin, vasopressin and prolactin in new breastfeeding mothers: Relationship to clinical characteristics and infant weight loss.	32	4–5 days postpartum	Suckling	Oxytocin
				Prolactin
				Vasopressin
				Milk volume
**IX: James et. al., 1995 [22]** Thirst induced by a suckling episode during breast feeding and its relation with plasma vasopressin, oxytocin and osmoregulation	10	28–52 weeks postpartum	Suckling	Oxytocin
			Non-suckling control period	Vasopressin
				Plasma osmolality
				Haematocrit
				Blood pressure
				Self-reported thirst
				Volume of water consumed
				Milk yield
**X: Johnston & Amico, 1986 [23]** A prospective longitudinal study of the release of oxytocin and prolactin in response to infant suckling in long term lactation	8	Two consecutive feeding sessions at: 2–4, 5–14 and 15–24 weeks postpartum	Suckling	Oxytocin
	3 non- breastfeeding		Formula feeding	Prolactin
				Vasopressin
**XI a: Jonas et. al., 2009 [24]** Effects of intrapartum oxytocin administration and epidural analgesia on the concentration of plasma oxytocin and prolactin, in response to suckling during the second day postpartum	61	2 days postpartum	Suckling	Oxytocin
			5 groups:	Prolactin
			• Controls without interventions (n = 20) • IV infusion of synthetic oxytocin in labour (n = 8) • IM injection of synthetic oxytocin postpartum (n = 13) • Epidural analgesia (n = 6)	ACTH
				XI b: Handlin et.al., 2009 [43]
				Cortisol
(XI a, XI b, XI c, XI d and XI d; articles based on the same material)				
				XI b: Handlin et.al., 2009 [43]
				Blood pressure
				XI c: Jonas et.al., 2008 [45]
				XI d: Handlin et al., 2012 [44]
			Epidural analgesia with IV infusion of synthetic oxytocin in labour (n = 14)	KSP
				XI e: Jonas et.al., 2008 [51]
**XII: Leake et.al., 1983 [25]** Oxytocin and prolactin responses in long-term breast-feeding	5	10–90, 90–180 and180 days-1 year postpartum	Breast stimulation with mechanical pump	Oxytocin
				Prolactin
				Progesterone

**XIII: Lucas et. al., 1980 [26]** Breast-feeding and plasma oxytocin concentrations	10	1–7 days postpartum	Suckling	Oxytocin
			Babies brought to mothers just before breastfeeding	Milk yield
**XIV: Matthiesen et. al., 2001 [27]** Postpartum maternal oxytocin release by newborns: effects of infant hand massage and suckling	10	Immediately postpartum	Skin-to-skin contact and suckling	Oxytocin
				Video recordings of interactions between mother and baby
			No pain relief or synthetic oxytocin in labour, birth or postpartum	
				Timing and amount of newborn hand massage of the breast and suckling
**XV: McNeilly et. al., 1983 [28]** Release of oxytocin and prolactin in response to suckling	10	4–6 days	Suckling	Oxytocin
		3–5 weeks and	Mothers and babies together before start of suckling	Prolactin
		10–11 weeks postpartum		Milk yield
**XVI: Mennella et. al., 2005 [29]** Acute alcohol consumption disrupts the hormonal milieu of lactating women	17	2–4 months postpartum	Breast stimulation with mechanical pump	Oxytocin
				Prolactin
				Cortisol
			Crossover design with control or drinking 0.4g/kg alcohol on two different days	Timing of milk ejection
				Milk yield
**XVII: Nissen et. al., 1995 [31]** Elevation of oxytocin levels early postpartum in women	18	Immediately postpartum	Skin-to-skin contact and suckling	Oxytocin
**XVIII a: Nissen et. al., 1996 [32]** Different patterns of oxytocin, prolactin but not cortisol release during breastfeeding in women delivered by caesarean section or by the vaginal route	37	2 days postpartum	Suckling	Oxytocin
			2 groups:	Prolactin
				Cortisol
				Blood pressure
				Timing of first interactions
			• Vaginal birth (n = 20) • Emergency caesarean section (n = 17)	Milk yield
				Duration (months) of exclusive and non-exclusive breastfeeding
**XVIII b: Nissen et. al., 1998 [30]** Oxytocin, prolactin, milk production and their relationship with personality traits in women after vaginal delivery or Cesarean section				KSP
(XVIII a and XVIII b; articles based on the same material)				
**XIX: Piron-Bossuyt et. al., 1978 [33]** Plasma oxytocin levels during lactation (French with English abstract)	8	4–8 days postpartum	Suckling	Oxytocin
**XX: Ueda et. al., 1994 [35]** Influence of psychological stress on suckling-induced pulsatile oxytocin release	22	5 days postpartum	Suckling with:	Oxytocin
			• Control: no stress (n = 8) • Mental stress (n = 7) • Noise stress (n = 7)	Prolactin
				Milk yield
**XXI a: Uvnäs Moberg et. al., 1990 a [37]** Oxytocin and prolactin levels in breast-feeding women. Correlation with milk yield and duration of breast-feeding.	55	4 days postpartum 3–4 months postpartum	• Suckling	Oxytocin
				Prolactin
				Somatostatin
				XXI c: Widstrom et. al., 1989 [46]
				XXI d: Widstrom et. al., 1991 [47]
**XXI b: Uvnäs Moberg et.al., 1990 b [36]** Personality traits in women 4 days postpartum and their correlation with plasma levels of oxytocin and prolactin				Birth weight
				Milk yield
				KSP
(XXI a, XXI b, XXI c and XXI d: articles based on the same material)				
**XXII: Velandia, 2012 [39]** Parent-infant skin-to-skin contact studies: Parent-infant interaction and oxytocin levels during skin-to-skin contact after Cesarean section and mother-infant skin-to-skin contact as treatment for breastfeeding problems	37	Immediately postpartum	Pre-labour caesarean section	Oxytocin
			All newborns had 5 min. skin-to- skin contact with mother then randomised to 25 min. skin-to-skin with mother (n = 17) or father (n = 20).	Oxytocin levels in fathers
				Newborn-parent tactile interactions
				Initiation of breastfeeding behaviours
				KSP 2 days postpartum
			19 women recieved additional 50 IU IV infusion of synthetic oxytocin postpartum over 2 hours	
**XXIII: Weitzman et. al., 1980 [38]** The effect of nursing on neurohypophyseal hormone and prolactin secretion in human subjects	12	2–3 days postpartum	Suckling	Oxytocin
				Prolactin
				Vasopressin
				Sodium
				Osmolality
**XXIV: Yokoyama et. al., 1994 [40]** Releases of oxytocin and prolactin during breast massage and suckling in puerperal women	12	3–5 days postpartum	Suckling (n = 6)	Oxytocin
			Breast massage (n = 6)	Prolactin
**XXV: Yuksel et.al., 2016 [42]** Immediate breastfeeding and skin-to-skin contact during cesarean section decreases maternal oxidative stress, a prospective randomized case-controlled study	90	Immediately postpartum	Prelabour caesarean section	Oxytocin
			Randomised to skin-to-skin and suckling immediately vs. at 1 hour postpartum	Postoperative pain scores
				Use of opioid analgesics
				Antioxidant status
				Oxidative stress indices.
			All women received IV infusion of synthetic oxytocin postpartum (20 IU per hour)	
**XXVI: Zinaman et. al., 1992 [41]** Acute prolactin and oxytocin responses and milk yield to infant suckling and artificial methods of expression in lactating women	23	28–42 days postpartum	Randomised to 15 min. in each of 5 groups	Oxytocin
				Prolactin
				Milk yield
			• Suckling • Hand expression • Breast pumping with electrical, battery or manual pump	

ACTH = adrenocorticotropic hormone, CRF = corticotropin releasing factor, T4 = thyroxine, IV = intravenous, IM = intramuscular, IU = international units, SOT = synthetic oxytocin, EDA = epidural analgesia, EPDS = Edinburgh Postnatal Depression Scale, KSP = Karolinska Scales of Personality, TSST = Trier Social Stress Test, STAI = State-Trait Anxiety Inventory, min. = minutes

**Table 3:** Oxytocin levels during breastfeeding or other types of breast stimulation.

Author, year and title	Timing of samples	Results	Method for analysis of oxytocin Basal and maximal oxytocin levels
**I: Amico & Finlay, 1986 [15]**	2 samples before stimulation and at 45 sec. after stimulation	In late-pregnant women oxytocin levels were significantly higher after mechanical breast stimulation than before (p<0.001)	RIA
			0.2–2 μU/mL (0.33–3.3 pg/mL)
Breast stimulation in cycling women, pregnant women and a woman with induced lactation: pattern of release of oxytocin, prolactin and luteinizing hormone		Oxytocin levels increased in response to breast stimulation in two of five cycling women (p<0.001, p<0.05)	
		Tactile and mechanical stimulation, as well as suckling of the breast in 1 adoptive mother, were associated with an increase in oxytocin above baseline	
**II: Amico et. al., 1994 [16]**	15 min. and 1 min. before and every 3 min. during 15 min. of breastfeeding	Breastfeeding was associated with episodic oxytocin release.	RIA
		Basal levels of oxytocin were 0.75 ± 0.06 μU/mL and increased to 2.81±1.30 μU/mL after 15 min. of breastfeeding (p = 0.0039)	0 to15 μU/mL (0–25.1 pg/mL)
Suckling-induced attenuation of plasma cortisol concentrations in postpartum lactating women			
		The size of the peaks varied between approx. 1.5–15 μU/mL	
**III: Chatterton et. al., 2000 [17]**	Continuous blood sampling every 10 min. starting 10 min. before and for 60 min. after start of pumping	Breast pumping induced no clear rise of oxytocin at 14, 28 and 42 days and oxytocin AUC did not change significantly over time	RIA
			35 pg/mL-70 pg/mL
Relation of plasma oxytocin and prolactin concentrations to milk production in mothers of preterm infants: influence of stress		At week 6, double pumping resulted in more than twice as much oxytocin across the 70-min. sampling compared to single pumping (p<0.001).	
		There was a correlation in each period between the oxytocin AUC and milk volume, which was significant at 6 weeks (p<0.043).	
**IV: Chiodera et.al., 1991 [18]**	2 min. before breastfeeding and during breastfeeding every second min. for 16 min.	Basal oxytocin levels were 2 pmol/L	RIA
		Breastfeeding induced a significant rise of oxytocin at 4, 6 and 8 min. compared with baseline levels (p< 0.01)	2–15 pg/mL
Relationship between plasma profiles of oxytocin and adrenocorticotropic hormone during suckling or breast stimulation in women			
		Oxytocin levels returned to baseline within 20 min.	
		Plasma levels of oxytocin remained constant in the sham group	
		Oxytocin levels obtained in the breastfeeding and control group differed significantly (p<0.01)	
		Mechanical breast stimulation in normal menstrual cycling women increased oxytocin levels significantly at 8 and 10 min. (p< 0.05 and 0.01 respectively)	
		Oxytocin levels returned to baseline with 20 min.	
		Plasma levels of oxytocin remained constant in the sham group	
**V: Christensson et. al., 1989 [19]**	Basal samples before and after examination of cervix	A clear rise of oxytocin levels was seen in response to nipple stimulation in most experiments in late-pregnant women	RIA
			0–300 pM (pg/mL)
Effect of nipple stimulation on uterine activity and on plasma levels of oxytocin in full term, healthy, pregnant women		15 min. after nipple stimulation was ended, oxytocin levels had returned to basal levels.	
	5 samples collected at 15 sec. intervals during each of 4–6 uterine contractions, during nipple stimulation and final sample at 15 min. after cessation of stimulation		
		9/10 women experienced uterine contractions	
		Levels of oxytocin during nipple stimulation increased significantly from the first contraction to subsequent contractions (p<0.01)	
		Peaks of oxytocin were seen that coincided in time with contractions	
**VI a: Cox et. al., 2015 [20]**	10 min. before and 1, 4, 7 and 10 min. during and at 10 min. after breastfeeding	Oxytocin levels in non-breastfeeding women were significantly lower than in breastfeeding women (p<0.01)	EIA
			2.5–8.5 pg/mL
		Women with depression/anxiety symptoms had lower levels of oxytocin during breastfeeding than women without symptoms (p< 0.05)	
Oxytocin and HPA stress axis reactivity in postpartum women			
		At 2 weeks, approximate mean oxytocin levels (estimated from graphs) were 4.5 pg/mL at baseline, peaking at 6–7 pg/mL over 3–10 min.	
**VI b: Stuebe et. al., 2013 [34]**		At 8 weeks, approximate mean oxytocin levels (estimated from graphs) were 2.5 pg/mL at baseline, peaking at 7–8.5 pg/mL over 3–10 min.	
Association between maternal mood and oxytocin response to breastfeeding (VI a and VI b; articles based on the same material)			
**VII: Dawood, et.al., 1981 [21]**	5 min. before and after the baby was brought to the mother	The mean plasma oxytocin level before suckling began was 10.8 ± 3.4 pg/mL and increased significantly to 22.4 ± 3.5 pg/mL 2 min. after suckling (p = 0.025)	RIA
			10.8–53.2 pg/mL
Oxytocin release and plasma anterior pituitary and gonadal hormones in women during lactation			
		Oxytocin levels reached a peak at 10 min. (mean level 53.2 ± 29.5 pg/mL) and then returned to basal at 20 min.	
	At 2, 5, 10, 15, 20, 25, and 30 min. after start of breastfeeding		
		A second peak occurred at 25 min. (28.2 ± 10.2 pg/mL) that was significantly higher than basal levels (p<0.05), perhaps in association with suckling of the second breast	
**VIII: Erickson et. al., 2019 [14]**	1 min. and 20 min. after start of breastfeeding	Oxytocin levels rose in significantly from 1 to 20 min. after start of suckling p<0.001	EIA
			1642–1714 pg/mL
Oxytocin, vasopressin and prolactin in new breastfeeding mothers: Relationship to clinical characteristics and infant weight loss.		Maternal oxytocin levels correlated with infant weight gain from birth to day 4–5 after birth in those who had o spontaneous labour. p = 0.002	
**IX: James et. al., 1995 [22]** Thirst induced by a	10 and 5 min. before, every 5 min. (max 8 samples) during breastfeeding and then two samples after breastfeeding	Peak oxytocin levels were significantly higher during breastfeeding (13.5 pmol/L) than during a control situation (5.7 pmol/L) (p<0.001)	RIA
			1–20 pmol/L (pg/mL)
suckling episode during breast feeding and its relation with plasma vasopressin, oxytocin and osmoregulation			
**X: Johnston & Amico, 1986 [23]**	Basal sample drawn at 15–1 min. before breastfeeding then every 3 min. during 15 min. of breastfeeding	Basal oxytocin levels were not significantly different between breastfeeding and non-breastfeeding women	RIA
			0.7–23.3 μU/mL (1.17–38.9)
A prospective longitudinal study of the release of oxytocin and prolactin in response to infant suckling in long term lactation		Oxytocin increased significantly in response to breastfeeding (p<0.00001) and remained elevated for 15 min.	
		There was no significant difference in the mean stimulated oxytocin levels or the pattern of release comparing the first and second feedings of the same day	
	Each woman participated in two breastfeeding experiment on the same day at 3 occasions		
		Peak oxytocin in response to breastfeeding was not significantly different in response to early (3.9±0.3 μU/mL), middle (4.5±0.3 μU/mL), and late (5.8±0.4 μU/mL) breastfeeding	
		In women who breastfed exclusively (n = 4), the stimulated oxytocin was significantly higher during late, vs. the early or middle breastfeeding period (p< 0.01)	
		In women who provided formula supplements (n = 4), oxytocin levels during breastfeeding did not change over the duration of lactation	
**XI: Jonas et. al., 2009 [24]**	4 samples at 30 sec. intervals before breastfeeding	Oxytocin levels were 131.6 pg/mL at 0 min. and 190.6 pg/mL at 1.5 min. after start of breastfeeding and returned to basal within 20 min. (women without medical interventions)	ELISA
			131,6–190,6 pg/mL
Effects of intrapartum oxytocin administration and epidural analgesia on the concentration of plasma oxytocin and prolactin, in response to suckling during the second day postpartum			
		3–4 pulses were observed within the first 10 min. of breastfeeding	
		The median oxytocin level based on all samples of each individual woman was 144.1 pg/mL	
	After onset of breastfeeding every 30 sec. during the first 7.5 min. and then at 10, 20, 30 and 60 min.		
	24 samples per woman		
**XII: Leake et. al., 1983 [25]**	At 3 min. intervals for 15 min. before pumping, then at 1, 2, 3, 6, 7, 8, 9, and 12 min. during and 6 min. after pumping	Oxytocin rose within 1 min. from basal levels in response to breast pumping at 10 days-3 months, 3–6 months and 6–12 months postpartum, and remained elevated for the duration of pumping (p<0.01)	RIA
			0.8–4.2 μU/mL (1,3–7.0 pg/mL)
Oxytocin and prolactin responses in long-term breast-feeding			
		There were no clear differences in basal or stimulated oxytocin levels at different times in lactation	
		There was no evident rise of oxytocin prior to pumping	
**XIII: Lucas et. al., 1980 [26]**	15 min. before start of breastfeeding, then consecutive samples at 20 sec. intervals during the first 10 min.	The release of oxytocin occurred in short peaks, the size of peaks varied from 0–123 ng/L (pg/mL)	RIA
			No extraction
Breast-feeding and plasma oxytocin concentrations		The total oxytocin response (AUC) was greater in multiparous than in primiparous women (p = 0.04)	0 to 123 ng/L (pg/mL).
		Similar amounts of oxytocin (AUC) were recorded in the beginning and at the end of the first week of breastfeeding	
		More milk was ingested by the babies at the end of the first week (21–66 mL range) in comparison with the first days after birth (0–9 mL)	
**XIV: Matthiesen et. al., 2001 [27]**	15 and 7 min. before birth, 7 min. after birth then every 15 min. during 2 hours after birth	Oxytocin levels varied over time, during the first 2 hours after birth. The mean of oxytocin levels was 56.6 fmol/mL	RIA
			Mean 56,6 fmol/mL (pg/mL)
Postpartum maternal oxytocin release by newborns: effects of infant hand massage and sucking		During skin-to-skin contact a coordinated pattern of infant hand and sucking movements was identified	
		Increased massage-like hand movements or sucking of the mother’s breast were followed by an increase in maternal oxytocin levels	
		The intensity of the stimulus was correlated with the rise of mothers’ oxytocin levels (p<0.005)	
**XV: McNeilly et. al., 1983 [28]**	15 min. before start of breastfeeding thereafter at one min. intervals from 10 min. before, until end of breastfeeding	Basal oxytocin values were 1.1 to 50.3 ng/L	RIA
		In all cases oxytocin increased in response to suckling, although this was more consistent at 4 and 11 weeks than immediately postpartum	1.1–59 ng/L (pg/mL)
Release of oxytocin and prolactin in response to suckling.			
		The pattern of oxytocin was pulsatile and the highest level varied between 11–59 ng/L (pg/mL)	
	One sample was collected 10 min. after breastfeeding	Oxytocin increased before suckling in all 10 women	
		In 5 of these women, the increase of oxytocin occurred just after the baby cried	
		There was no correlation between the amount of oxytocin released and parity of the mother, or the volume of milk taken at the feed by test weighing	
**XVI: Mennella et. al., 2005 [29]**	40, 25, 10 min. before drinking then at approx. 35 mins. after drinking and every 2 min. for 16 min. during breast pumping then every 15 min. after cessation of pumping up to 90 min.	Oxytocin rose in response to breastfeeding	RIA
		Peak levels of oxytocin on the day women consumed alcohol were not significantly different from peak levels in the control day	(no extraction)
Acute alcohol consumption disrupts the hormonal milieu of lactating women			20 to 40 pg/mL
		Oxytocin AUC was significantly smaller during alcohol consumption (p = 0.005)	
		Oxytocin levels during the initial min. of breast stimulation correlated negatively with milk ejection latency, on the day women consumed alcohol (p = 0.02)	
		The oxytocin responses of individual women correlated between experiments with and without alcohol	
		Women (12/17) who produced less oxytocin on the alcohol day also had lower milk yields during pumping, compared with the remaining (5) women (p = 0.008)	
**XVII: Nissen et. al., 1995 [31]**	15 and 7 min. before birth, 7 min. after birth then every 15 min. during 2 hours after birth	Oxytocin levels rose significantly from 20 pM before breastfeeding to 47, 34 and 40 pM at 15, 30 and 45 min. during skin- to- skin contact immidiately after birth (p = 0.007, 0.02 and 0.02 respectively) and returned to basal levels at 60 min.	RIA
			20 to 47 pM (pg/mL)
Elevation of oxytocin levels early postpartum in women			
		The initial rise of oxytocin coincided with placental expulsion, which occurred at median 10.5 min. postpartum	
		No clear rise of oxytocin was observed in conection with the first breastfeeding, which occured in a median of 87 min.	
**XVIII: Nissen et. al., 1996 [32]**	Observational data collected in relation to birth	Basal oxytocin levels before breastfeeding were 12 pmol/L (Q25-Q75: 10–20) in the vaginal birth group	RIA
			7–118 pmol/L (pg/mL)
Different patterns of oxytocin, prolactin but not cortisol release during breastfeeding in women delivered by caesarean section or by the vaginal route		Breastfeeding was associated with a pulsatile oxytocin release and 0–5 pulses occurred during 10 min. of breastfeeding	
	Breastfeeding data collected at 2 days after birth		
		14/20 women had at least one pulse	
		The number of pulses during the first 10 min. correlated with the duration of exclusive breastfeeding (p = 0.006)	
	4 samples at 30 sec. intervals before onset of breastfeeding		
		The number of oxytocin pulses correlated with milk yield (p = 0.03)	
**XVIII b: Nissen et. al., 1998 [30]**	After onset of breastfeeding, every 30 sec. during the first 7.5 min. and then at 10, 20, 30 and 60 min.		
Oxytocin, prolactin, milk production and their relationship with personality traits in women after vaginal delivery or cesarean section			
	24 samples per woman		
(XVIII a and XVIII b; articles based on the same material)			
**XIX: Piron-Bossuyt et. al., 1978 [33]**	Samples were collected at 5, 0, 5, 10, 15 and 20 min. in relation to breastfeeding	Oxytocin levels rose from basal levels 8–85 μU/mL to 10–129 μU/mL during breastfeeding	RIA
			8 to 152 μU/mL (13.4–254 pg/mlL
[Plasma oxytocin levels during lactation]			
**XX: Ueda et. al., 1994 [35]**	Samples were collected at 2 min. intervals from 10 min. before and for 20 min. during breastfeeding	In the control group, basal oxytocin was 2±1.51 pg/mL	RIA
		A pulsatile pattern of oxytocin was induced by breastfeeding, with mean pulse amplitude 9.1±1.3 pg/mL	1.69 ±0.54 to 9.1±1.3 pg/mL
Influence of psychological stress on suckling-induced pulsatile oxytocin release			
		The number of pulses over 20 min. was significantly lower in the mental stress (1.28±0.76) and noise stress (1.14±0.38) groups compared with the control group (2.25±0.71) (p< 0.05 and <0.01 respectively). Pulse amplitudes were not significantly different.	
**XXI a: Uvnäs Moberg et. al., 1990 a [37]**	6 basal samples were collected 30 sec. before the baby was brought to mother	Basal oxytocin levels at 3–4 months were around 50% of those at 4 days postpartum	RIA
			15–45 pg/mL
Oxytocin and prolactin levels in breast-feeding women. Correlation with milk yield and duration of breast-feeding		Oxytocin levels increased in response to breastfeeding at 4 days and 3–4 months postpartum (p<0.01 and p<0.05)	
	6 samples every 30 sec. after initiation of breastfeeding and then at 10, 12, 13, 20, 30 and 60 min.		
		Average oxytocin levels were twice as high in response to breastfeeding at 3–4 months postpartum compared to 4 days postpartum	
**XXI b Uvnäs Moberg et. al., 1990 b [36]**			
	18 samples per woman		
		Basal as well as stimulated individual oxytocin levels obtained 4 days and 3–4 months postpartum correlated significantly for each individual woman	
Personality traits in women 4 days postpartum and their correlation with plasma levels of oxytocin and prolactin			
		At 2 and 12 months after weaning, mean basal oxytocin levels were 30pM and 10pM respectively, significantly lower than at 4 days postpartum (p<0.001 for both)	
(XXI a, XXI b, XXI c and XXI d: articles based on the same material)		At 6 months, 20% of women were exclusively and 60% were partially breastfeeding	
		Women who breastfed exclusively at 3–4 months had significantly higher basal oxytocin levels than those partially breastfeeding.	
		Milk yield was 55±25 mL at 4 days (n = 47) and 140±40 mL at 4 months postpartum (n = 25)	
		Milk yield did not correlate with oxytocin before or during breastfeeding	
		There was a significant correlation between oxytocin levels at 3–4 months and infant birth weight (p = 0.048)	
**XXIII: Weitzman et. al., 1980 [38]**	0 min. before suckling, every 3 min. for 15 min. during breastfeeding and 5 min. after breastfeeding	Oxytocin levels increased significantly during breastfeeding from 1.1±0.2 μU/mL to 3.6±0.6 μU/mL at 3 min. (p<0.001), peaking at 6 min. (6.4±1.5 μU/mL) (p<0.005)	RIA
			1.1 to 6.4 μU/mL (1.8–10.7 pg/mL)
The effect of nursing on neurohypophyseal hormone and prolactin secretion in human subjects			
		Oxytocin levels remained elevated during the 15 min. of breastfeeding and returned to basal levels 5 min. after cessation	
**XXIV: Yokoyama et. al., 1994 [40]**	Every second min. from 10 min. before the start of breastfeeding/breast massage until the end of breastfeeding/breast massage (after 20 min.)	Basal levels of oxytocin were 1.9±0.18 pg/mL	RIA
		Breastfeeding induced a pulsatile release of oxytocin	1.9–40 pg/mL
		The mean number of pulses during the 20-min. breastfeeding period was 2.5±0.19 and average size was around 14 pg/mL	
Releases of oxytocin and prolactin during breast massage and suckling in puerperal women			
		During breast massage, oxytocin levels increased and remained high	
		The release was not pulsatile, but rather a sustained elevation	
		There was a significantly greater increase in mean oxytocin levels following breast massage (11.06±1.41 pg/mL) compared to suckling (2.19±0.47 pg/mL)	
**XXVI Zinaman et. al., 1992 [42]**	15, 0, 10, 20, 30, 50, and 60 min. after start of suckling, hand expression, or electrical pumping (3 types of machines)	Oxytocin levels (given as AUC) were highly variable and increased with each method, with no significant differences between groups	RIA
			Values are presented as AUC
Acute prolactin and oxytocin responses and milk yield to infant suckling and artificial methods of expression in lactating women		In the suckling group, several women exhibited their peak values just prior to initiation of suckling, which was not seen with the artificial methods	

AUC = area under the curve, Q25-Q75 = interquartile range, RIA = radioimmunoassay, ELISA = enzyme-linked immunosorbent assay (also called EIA = enzyme immunoassay), IV = intravenous, IM = intramuscular, IU = international units, μU/mL = microUnits per millilitre, ng/L = nanograms per litre, pM = picoMolar = pmol/L = picomoles per litre, pg/mL = picogram per millilitre, fmol/mL = femtomoles per millilitre. vs. = versus, min. = minutes, sec. = seconds, approx. = approximately

Note that pg/mL = ng/L, fmol/mL = pmol/L = pM, pg/mL = pmol/L, 1 μU/mL = 1 pg/L times 1.67.

**Table 4:** Oxytocin levels during breastfeeding or other types of /breast stimulation, women with medical interventions during labour, birth or postpartum.

Author, year and title	Timing of samples	Results	Method of analysis of oxytocin levels Range of oxytocin levels
**Emergency caesarean section**			
**XVIII a: Nissen et. al., 1996 [32]**	Observational data collected in relation to birth	Following emergency caesarean section, women first saw, held, and breastfed their babies at medians of 90, 120 and 240 min. respectively	RIA
			Extraction of samples
Different patterns of oxytocin, prolactin but not cortisol release during breastfeeding in women delivered by caesarean section or by the vaginal route		These events occurred significantly later than after vaginal birth (p = 0.0001, 0.0001, 0.0026 respectively)	7–118 pmol/L approx. for all women VD and CS
	Breastfeeding data collected at 2 days after birth		
		Basal oxytocin levels were 18 pmol/L (Q25-75: 15–25 pmol/L) among women breastfeeding 2 days after emergency caesarean section	
		Pulses of oxytocin were observed during the first 10 min. of breastfeeding	
	4 blood samples at 30 sec. intervals before breastfeeding	The mean number of pulses was significantly lower compared to vaginal birth (p = 0.002), with 3/17 women having ≥ 1 pulse vs. 14/20 following vaginal birth (p = 0.004)	
**XVIII b: Nissen et. al., 1998 [30]**			
	After onset of breastfeeding every 30 sec. during the first 7.5 min. and then at 10, 20, 30 and 60 min.	In regression analysis, mode of birth, age at first suckling and the KSP variable somatic anxiety had the greatest influence on the number of pulses (p = 0.0065, 0.022, and 0.03 respectively)	
Oxytocin, prolactin, milk production and their relationship with personality traits in women after vaginal delivery or Cesarean section			
		Vaginal birth, early suckling and low levels of somatic anxiety were associated with more pulses	
		The number of oxytocin pulses in response to breastfeeding correlated positively with milk yield (p = 0.03) in the whole group of women (caesarean and vaginal birth)	
(XVIII a and XVIII b; articles based on the same material)	24 samples per woman		
**Prelabour caesarean section**			
**XXII: Velandia 2012 [39]**	1 sample at 5 min. before birth.	Oxytocin rose significantly over the first 2 hours postpartum for mothers in both skin-to-skin and control groups compared to basal levels (p<0.001)	RIA
			20 to 65 pM
Parent-infant skin-to-skin contact studies: Parent-infant interaction and oxytocin levels during skin-to-skin contact after cesarean section and mother-infant skin-to-skin contact as treatment for breastfeeding problems	After birth every 5 min. during the first 45 min. (11 samples) and then every 15 min. to 2 hours postpartum (5 samples)	There was a small but significant rise among fathers (p = 0.008)	
		No significant difference in oxytocin between mothers with or without skin-to-skin contact	
		Women who received IV infusion of synthetic oxytocin postpartum had significantly higher oxytocin levels during skin-to-skin contact and suckling compared to women without infusion (p = 0.023)	
	16 samples per woman		

**XXV: Yuksel et.al., 2016 [42]**	2 samples before spinal anaesthetic and 15 min. after caesarean was completed	Following prelabour caesarean section, oxytocin was higher with immediate newborn skin-to-skin contact and suckling vs. separation from newborn (p = 0.003).	ELISA
			187–890 pg/mL
Immediate breastfeeding and skin-to-skin contact during cesarean section decreases maternal oxidative stress, a prospective randomized case-controlled study			
		Mothers with skin-to-skin contact had significantly better oxidative stress indices (p<0.001) and antioxidant status (p<0.001)	
		Oxytocin levels postpartum positively correlated with better oxidative stress indices and antioxidant status (p<0.001).	
**Medical interventions: epidural analgesia and synthetic oxytocin**			
**XI a: Jonas et. al., 2009 [24]**	Breastfeeding data collected at 2 days after birth	**Epidural analgesia (EDA**)	
		Oxytocin levels were (median) 166.2 pg/mL at 0 min. and 211.2 pg/mL at 1.5 min. after start of breastfeeding	
Effects of intrapartum oxytocin administration and epidural analgesia on the concentration of plasma oxytocin and prolactin, in response to suckling during the second day postpartum			
		Median oxytocin level during breastfeeding in this group was 183.8 pg/mL	
	4 samples at 30 sec. intervals before breastfeeding		
	After onset of breastfeeding every 30 sec. during the first 7.5 min. and then at 10, 20, 30 and 60 min.	**Intravenous infusion of synthetic oxytocin in labour (SOT IV)**	
		Oxytocin levels were (median) 156.3 pg/mL at 0 min. and and176.6 pg/mL at 1.5 min. after start of breastfeeding	
		Median oxytocin level during breastfeeding in this group was 171.3pg/mL	
		The higher the mothers’ oxytocin in response to suckling, the shorter the duration of skin-to-skin contact was before the initiation of breastfeeding during this episode (p = 0.023)	
	24 samples per woman		
		**Epidural analgesia with intravenous infusion of synthetic oxytocin in labour (EDA/SOT IV)**	
		Oxytocin levels were 96.2 pg/mL at 0 min. and 121.4 pg/mL at 1.5 min. after start of breastfeeding	
		The median oxytocin level during breastfeeding in this group was 106.8 pg/mL, which was significantly lower compared to other groups (SOT, p = 0.005; SOT IM, p = 0.033; EDA, p = 0.051)	
		The higher the total dose of intravenous infusion of synthetic oxytocin during labour, the lower the women’s median oxytocin level was during a breastfeeding episode 2 days after birth (p = 0.019)	
		**Intramuscular injection of synthetic oxytocin postpartum (SOT IM)**	
		Oxytocin levels were 159.3 pg/mL at 0 min. and 173.0 pg/mL at 1.5 min. after start of breastfeeding	
		The median oxytocin level during breastfeeding in this group was158.2 pg/mL	
		**The whole group, without and with medical interventions**	
		Median oxytocin levels were 120.0 pg/mL at 0 min. and 166.0 pg/mL at 1.5 min. after start of breastfeeding (p = 0.001)	
		A pulsatile pattern was recorded for the first 10 min., with 3–4 pulses observed	
		The median oxytocin level during breastfeeding in this group was 144.3 pg/mL	

Q25-Q75 = interquartile range RIA = radioimmunoassay, ELISA = enzyme-linked immunosorbent assay (also called EIA = enzyme immunoassay), IV = Intravenous, IM = intramuscular, SOT = synthetic oxytocin, EDA = epidural analgesia, pM = picoMolar, pmol/L = picomoles per litre, pg/mL = picogram per millilitre, KSP = Karolinska Scales of personality, sec. = seconds, min. = minutes, vs. = versus, approx. = approximately

Note that pg/mL = ng/L, fmol/mL = pmol/L = pM, pg/mL = pmol/L, 1 μU/mL = 1 pg/L times 1.67.

**Table 5:** Prolactin levels during breastfeeding or other types of /breast stimulation, including women with medical interventions during labour, birth or postpartum.

**WITHOUT MEDICAL INTERVENTIONS**	
**Author, year and title**	**Results**
**I: Amico & Finlay, 1986 [15]**	Prolactin levels did not increase in response to nipple stimulation in pregnant women in the third trimester
Breast stimulation in cycling women, pregnant women and a woman with induced lactation: pattern of release of oxytocin, prolactin and luteinizing hormone	
	Prolactin increased in response to suckling at 5 and 40 days of lactation in one women with induced lactation
**III: Chatterton et. al., 2000 [17]**	Prolactin levels rose during mechanical pumping for preterm infants at 2 (p<0.001) and 4 weeks (p<0.001) but not at 6 weeks
Relation of plasma oxytocin and prolactin concentrations to milk production in mothers of preterm infants: influence of stress	
	Prolactin response (AUC) declined significantly between weeks 2 and 6 weeks postpartum (p = 0.03)
	After double pumping, prolactin was higher in comparison with single pumping (p = 0.002) but no significant differences in milk volume
	No correlation between prolactin and milk volume
**VII: Dawood, et. al., 1981 [21]**	Significant rise in prolactin 10 min. after initiation of breastfeeding (p = 0.025)
Oxytocin release and plasma anterior pituitary and gonadal hormones in women during lactation	Prolactin levels remained elevated for 30 min.
**VIII: Erickson et. al., 2019 [14].**	Significant rise in prolactin at 20 min. after start of breastfeeding p = 0.009
Oxytocin, vasopressin and prolactin in new breastfeeding mothers: Relationship to clinical characteristics and infant weight loss.	
**X: Johnston & Amico, 1986 [23]**	Prolactin levels rose in response to breastfeeding with a significant peak at 15 min. (p<0.003)
A prospective longitudinal study of the release of oxytocin and prolactin in response to infant suckling in long term lactation	Basal prolactin decreased significantly from 1 to 24 weeks postpartum (p<0.05)
**XI a: Jonas et. al., 2009 [24]**	In the control group (without medical interventions), there was a non-significant rise of prolactin in response to breastfeeding
Effects of intrapartum oxytocin administration and epidural analgesia on the concentration of plasma oxytocin and prolactin, in response to suckling during the second day postpartum	
**XI: Leake et.al., 1983 [25]**	During mechanical breast pumping in long-term breastfeeding women, basal prolactin was non-significantly higher at 6–12 months than at 10 days-3 months and 3–6 months
Oxytocin and prolactin responses in long-term breast-feeding	
	No significant rise of prolactin during breast pumping
**XVI: McNeilly et.al., 1983 [28]**	Sustained prolactin release in response to breastfeeding in 8/10 women
Release of oxytocin in response to breastfeeding	No release of prolactin before suckling in the presence of the baby
	No correlation between prolactin and milk volume
**XVIII a: Nissen et. al., 1996 [32]**	Significant rise of prolactin at 20 and 30 min. after breastfeeding initiation (p = 0.002 and p = 0.0008, respectively) for women following vaginal birth
Different patterns of oxytocin, prolactin but not cortisol release during breastfeeding in women delivered by caesarean section or by the vaginal route	
**XVIII b: Nissen et. al., 1998 [30]**	
Oxytocin, prolactin, milk production and their relationship with personality traits in women after vaginal delivery or Cesarean section	
(XVIII a and XVIII b; articles based on the same material)	
**XX: Ueda et. al., 1994 [35]**	Prolactin levels rose during breastfeeding in all groups, with and without stress (noise and mental calculation)
Influence of psychological stress on suckling-induced pulsatile oxytocin release	No significant effect on prolactin release from mental calculation or noise
	Similar milk yield in the 3 groups
**XXI a: Uvnäs Moberg et. al., 1990 a [37]**	Basal levels of prolactin were significantly higher after birth than at 3–4 months
	Twofold rise of prolactin levels 10 min. after start of breastfeeding at 3–4 days and 3–4 months
	The increase in prolactin levels (AUC) was related to basal prolactin at 3–4 months (p = 0.001)
Oxytocin and prolactin levels in breast-feeding women. Correlation with milk yield and duration of breast-feeding	No correlation between prolactin AUC at 3–4 days and 3–4 months
	Significant decrease of prolactin within 24 h of weaning (p<0.05)
	Significant decrease of prolactin 2 and 12 months after weaning vs. 3–4 days postpartum (p = 0.001)
	Prolactin at 3–4 months correlated to the remaining period of breastfeeding
	Prolactin was significantly higher at 3–4 months in exclusive breastfeeding mothers vs. supplementary feeding
	No significant relation between prolactin and milk yield at 3–4 days and 3–4 months
**XXIII: Weitzman et. al., 1980 [38]**	Significant rise of prolactin at 15 min. after breastfeeding initiation (p<0.05)
The effect of nursing on neurohypophyseal hormone and prolactin secretion in human subjects	
	No rise of prolactin in breastfeeding women who did not breastfeed at this time
**XXIV: Yokoyama et. al., 1994 [40]**	Significant rise of prolactin levels in response to breastfeeding (p = 0.05)
Releases of oxytocin and prolactin during breast massage and suckling in puerperal women	No significant rise of prolactin levels in response to breast massage
**XXVI Zinaman et. al., 1992 [41]**	Significant rise of prolactin in response to suckling, hand expression, and using manual, battery or electric pumps
Acute prolactin and oxytocin responses and milk yield to infant suckling and artificial methods of expression in lactating women	Significantly higher prolactin levels with breastfeeding or a double electric pump vs. consecutive single-breast pumping using a battery-power or manual pump, or hand expression (p = 0.05)
**WITH MEDICAL INTERVENTIONS**	
**Emergency caesarean section**	
**XVIII a: Nissen et. al., 1996 [32]**	Basal prolactin levels were significantly higher in the caesarean section group than in the vaginal birth group (p = 0.03)
Different patterns of oxytocin, prolactin but not cortisol release during breastfeeding in women delivered by caesarean section or by the vaginal route	Breastfeeding induced no rise of prolactin from 0 to 20 or 30 min. in the caesarean section group
	Prolactin levels fell significantly from 0 to 60 min. breastfeeding in the caesarean section group (p = 0.026)
	No significant differences in breastfeeding-induced prolactin levels between the caesarean vs. vaginal birth groups
	In relation to the ratios between basal prolactin and levels at 20 and 30 min.:
**XVIII b: Nissen et. al., 1998 [30]**	
Oxytocin, prolactin, milk production and their relationship with personality traits in women after vaginal delivery or Cesarean section	• Significantly fewer women in caesarean group (7) vs. vaginal birth (17) showed an elevation of this ratio at 30 min. (p = 0.015) • Significantly smaller ratio at 20 min. in caesarean group vs. vaginal birth groups (p = 0.001)
(XVIII a and XVIII b; articles based on the same material)	
	In regression analysis, the prolactin pattern was significantly influenced by mode of birth (p = 0.015) (prolactin levels rose more in response to breastfeeding following vaginal birth vs. caesarean) and duration of the breastfeeding session (p = 0.038) (the longer the breastfeeding session, the higher the prolactin levels)
	In the whole group of women, basal levels of prolactin correlated with basal levels of oxytocin (p = 0.04)
**Other types of medical interventions**	
**X: Jonas et. al., 2009 [24]**	**Epidural analgesia (EDA)**
Effects of intrapartum oxytocin administration and epidural analgesia on the concentration of plasma oxytocin and prolactin, in response to suckling during the second day postpartum	No significant rise of prolactin in response to breastfeeding at 10 min.
	Significant negative correlations between oxytocin and prolactin levels at 20 and 60 min. (p = 0.019 for both)
	**Intravenous infusion of synthetic oxytocin in labour (SOT IV)**
	Significant rise of prolactin at 10 min. (p = 0.012)
	The rise of prolactin at 10 min. was significantly higher vs. control group (p = 0.006)
	Prolactin was still significantly elevated 60 min. after breastfeeding (p = 0.0012)
	**Epidural analgesia with intravenous infusion of synthetic oxytocin in labour (EDA/SOT IV)**
	Significant rise of prolactin at 10 min. (p = 0.008)
	The rise of prolactin at 10 min. was significantly higher in EDA/SOT vs. control group (p = 0.003)
	Prolactin was still significantly elevated 60 min. after breastfeeding (p = 0.0023)
	Significant positive correlation between oxytocin and prolactin at 0–20 min. (p = 0.035)
	**Intramuscular injection of synthetic oxytocin postpartum (SOT IM)**
	No significant rise of prolactin at 10 min. in response to breastfeeding
	Several positive significant correlations between single oxytocin and prolactin levels (p = 0.004)

**Table 6:** Stress indicators during breastfeeding or other types of /breast stimulation, including women with medical interventions during labour, birth or postpartum.

**WITHOUT MEDICAL INTERVENTIONS**	
**II: Amico et al., 1994 [16]**	Significant decrease in cortisol levels at 15 min. after start of breastfeeding (p = 0.001)
Suckling-induced attenuation of plasma cortisol concentrations in postpartum lactating women	No decrease in cortisol levels at 15 min. after start of formula feeding (n = 2)
**III: Chatterton et. al., 2000 [17]**	Salivary cortisol at 30 min. after start of mechanical breast pumping was significantly lower at 6 weeks compared to 0 weeks after birth (p = 0.028)
Relation of plasma oxytocin and prolactin concentrations to milk production in mothers of preterm infants: influence of stress	
	Salivary amylase at 30 min. after start of mechanical breast pumping was significantly lower at 6 weeks compared to 0 weeks after birth (p = 0.045)
**IV: Chiodera et.al., 1991 [18]**	ACTH levels significantly decreased from 0 to 6 min. after breastfeeding initiation (p<0.05)
Relationship between plasma profiles of oxytocin and adrenocorticotropic hormone during suckling or breast stimulation in women	Cortisol levels significantly decreased from 0 to 8 min. after breastfeeding initiation (p<0.05)
	The nadir of ACTH coincided with the peak value of oxytocin at 6 min. during suckling
	Significant negative correlation between oxytocin and ACTH during suckling (p<0.001)
	This effect differed significantly from non-suckling controls (p<0.05)
	With mechanical breast stimulation in normal cycling women, ACTH levels significantly decreased from 0 to 6 min. after initiation (p<0.05)
	With mechanical breast stimulation in normal cycling women, cortisol levels significantly decreased from 0 to 10 min. after initiation (p<0.05)
	No effect on either ACTH or cortisol in controls
	With mechanical breast stimulation in normal cycling women, the nadir of ACTH coincided with peak value of oxytocin at 10 min.
	With mechanical breast stimulation in normal cycling women, there was a significant negative correlation between oxytocin and ACTH (p<0.001)
**VI a: Cox et. al. 2015 [20]**	Women with symptoms of postpartum depression had higher cortisol levels at baseline and during breastfeeding, compared to women without such symptoms (p< 0.05)
Oxytocin and HPA stress axis reactivity in postpartum women	
	In women with and without symptoms of postpartum depression, cortisol levels decreased in response to breastfeeding
**VI b: Stuebe et. al., 2013 [34]**	
	After the end of the breastfeeding session, cortisol levels in women (with and without postpartum depression) were non-reactive to stress (TSST test) compared to non-breastfeeding women, whose cortisol levels rose (p<0.1)
Association between maternal mood and oxytocin response to breastfeeding	
(VI a and VI b; articles based on the same material)	After the end of the breastfeeding session, heart rate responses to stress (TSST test) in women with and without postpartum depression were lower, compared to non-breastfeeding women (p<0.01)
**VIII: Erickson et. al., 2019 [14].**	Significant decrease of vasopressin levels from 1 to 20 min. after start of breastfeeding p = 0.05
Oxytocin, vasopressin and prolactin in new breastfeeding mothers: Relationship to clinical characteristics and infant weight loss.	Note that vasopressin levels were measured by EIA
**XI b: Handlin et. al 2009 [43]**	In the control group (no medical interventions):
Effects of suckling and skin-to-skin contact on maternal ACTH and cortisol levels during the second day postpartum-influence of epidural analgesia and oxytocin in the perinatal period	Significant decrease of ACTH in response to breastfeeding (p = 0.044)
	Significant decrease in cortisol levels in response to breastfeeding (p = 0.0001)
	Significant negative correlation was found between oxytocin variance and median ACTH levels (p = 0.41)
	Significant relationship between median ACTH and median cortisol levels (p = 0.0013)
**XI d: Handlin et. al 2012 [44]**	
Influence of common birth interventions on maternal blood pressure patterns during breastfeeding 2 days after birth	Both systolic and diastolic blood pressure fell significantly from base to 60 min. after breastfeeding initiation in the control group (p = 0.001 and p = 0.004 respectively)
	Over 25 weeks of lactation, there was a significant fall in systolic (15 mm Hg, p = 0.001) and diastolic blood pressure (10mm Hg p = 0.002)
**XI c: Jonas et.al., 2008 [45]**	
Short and long-term decrease of blood pressure in women during breastfeeding	
	Both systolic and diastolic blood pressure fell significantly during breastfeeding in the control group (p = 0.001 and p = 0.004)
(XI a, XI b, XI c, XI d and XI d; articles based on the same material)	
**XVIII a: Nissen et. al., 1996 [32]**	Cortisol levels fell significantly from 20 to 60 min. after the onset of breastfeeding in the vaginal birth group (p = 0.002)
Different patterns of oxytocin, prolactin but not cortisol release during breastfeeding in women delivered by caesarean section or by the vaginal route	Blood pressure fell significantly from the onset of breastfeeding to 60 min. later in the vaginal birth group (p = 0.025)
**XVIII b: Nissen et. al., 1998 [30]**	
Oxytocin, prolactin, milk production and their relationship with personality traits in women after vaginal delivery or Cesarean section	
(XVIII a and XVIII b; articles based on the same material)	
**WITH MEDICAL INTERVENTIONS**	
**Emergency caesarean section**	
**XVIII a: Nissen et. al., 1996 [32]** Different patterns of oxytocin, prolactin but not cortisol release during breastfeeding in women delivered by caesarean section or by the vaginal route	Cortisol levels fell in response to breastfeeding in the caesarean section group, which was significant at 7.5 min. (p = 0.0042)
	Systolic blood pressure fell non-significantly in the caesarean section group at 60 min. (p = 0.062)
	The fall of blood pressure was steeper in the caesarean section group than in the vaginal birth group, but no significant differences were found between the groups
**XVIII b: Nissen et. al., 1998 [30]** Oxytocin, prolactin, milk production and their relationship with personality traits in women after vaginal delivery or Cesarean section	
(XVIII a and XVIII b; articles based on the same material)	
**Elective caesarean section**	
**XXV: Yuksel et.al. 2016 [42]**	Significantly better maternal oxidative stress indices and antioxidant status with immediate newborn skin-to-skin contact and suckling vs. early separation from newborn (p<0.001)
Immediate breastfeeding and skin-to-skin contact during cesarean section decreases maternal oxidative stress, a prospective randomized case-controlled study	
	Significant positive correlation between postpartum oxytocin and better oxidative stress indices and antioxidant status (p<0.001)
	Non-significant reductions in postoperative pain score and use of opioid analgesic for women with immediate vs. delayed contact
**Other medical interventions**	
**XI b: Handlin et. al 2009 [43]**	**Epidural analgesia (EDA)**
Effects of sucking and skin-to-skin contact on maternal ACTH and cortisol levels during the second day postpartum-influence of epidural analgesia and oxytocin in the perinatal period	ACTH levels decreased non-significantly in response to breastfeeding in the EDA group
	Basal cortisol levels were significantly lower in the EDA group compared with the EDA/SOT IV group (p = 0.033)
	Median cortisol levels were significantly lower in the EDA group compared to EDA/SOT IV group (p = 0.041)
	Significant negative correlation between oxytocin variability and median ACTH levels (p = 0.005)
	**Intravenous infusion of synthetic oxytocin in labour (SOT IV)**
**XI d: Handlin et. al 2012 [44]**	Non-significant decrease in ACTH levels in response to breastfeeding
Influence of common birth interventions on maternal blood pressure patterns during breastfeeding 2 days after birth	Non-significant decrease in cortisol levels in response to breastfeeding
	**Epidural analgesia with intravenous infusion of synthetic oxytocin in labour (EDA/SOT IV)**
	Non-significant decrease of ACTH levels in response to breastfeeding
(XI a, XI b, XI c, XI d and XI d; articles based on the same material)	Significant decrease in cortisol levels in response to breastfeeding (p = 0.028)
	**Intramuscular injection of synthetic oxytocin postpartum (SOT IM)**
	Non-significant decrease in ACTH levels in response to breastfeeding
	Non-significant decrease in cortisol levels in response to breastfeeding
	Duration of suckling correlated negatively with median ACTH in SOT IM group (p = 0.031)
	Duration of skin to skin contact correlated with decrease of cortisol levels during the first 2.5 min. of suckling (p = 0.004)
	**The whole group, without and with medical interventions**
	ACTH levels decreased significantly after 60 min. of breastfeeding (p = 0.001)
	Cortisol levels decreased significantly after 60 min. of breastfeeding (p = 0.0001)
	Significant negative correlation between median oxytocin and median ACTH levels (p = 0.009)
	Significant negative correlation between oxytocin variance and median ACTH levels (p = 0.037)
	No significant correlation between median oxytocin levels or oxytocin variance and cortisol levels
	Significant positive correlation between median ACTH and cortisol levels
	Significant negative correlation between the duration of suckling and median ACTH levels (p = 0.041)
	No significant relationship between the duration of suckling and median cortisol levels
	No significant relationship between the duration of skin to skin contact and median ACTH levels
	Significant negative correlation between the duration of skin to skin contact and cortisol levels (p = 0.044)
	No significant relationship between ACTH and cortisol in interventions
	**Epidural analgesia (EDA)**
	Basal diastolic blood pressure was significantly lower vs. control (p = 0.045); SOT IV (p = 0.041); and EDA/SOT IV (p = 0.024)
	No significant fall of systolic or diastolic blood pressure with breastfeeding
	**IV infusion of synthetic oxytocin in labour (SOT IV)**
	The decrease in diastolic blood pressure with breastfeeding approached significance (p = 0.05)
	No effect on systolic blood pressure
	**Epidural analgesia with IV infusion of synthetic oxytocin in labour (EDA/SOT IV)**
	Both systolic and diastolic blood pressure fell significantly (p = 0.028 and p = 0.002 respectively)
	**Intramuscular injection of synthetic oxytocin postpartum (SOT IM)**
	Both systolic and diastolic blood pressure fell significantly (p = 0.006 and p = 0.001 respectively)
	Duration of skin-to-skin contact pre-suckling correlated with decrease in systolic blood pressure from 0 to 60 min. (p = 0.046)

**Table 7:** Nutritional and metabolic variables in connection with breastfeeding.

Study	Summary of data
**IX: James et. al., 1995 [22]**	The sensation of thirst rose when breastfeeding was initiated
Thirst induced by a suckling episode during breast feeding and its relation with plasma vasopressin, oxytocin and osmoregulation	Thirst ratings were significantly higher during breastfeeding than during a control situation (p = 0.013)
	The peak level of thirst correlated with the volume of water consumed to satiety following the breastfeeding period (p = 0.024)
	The increase in the sensation of thirst seemed to be parallel with the rise of oxytocin during breastfeeding
	No changes in osmolality, haematocrit, blood pressure or vasopressin were observed in response to breastfeeding
**XXI c: Widström et. al., 1989 [46]**	Somatostatin levels tended to fall in response to suckling at 4 days after birth and tended to rise at 3–4 months
	The rise at 3–4 months was significantly higher than at 4 days (p = 0.01)
Maternal somatostatin levels and their correlation with infant birth weight	There was a strong correlation between average somatostatin levels obtained at the two occasions (p = 0.0001)
	Maternal somatostatin levels obtained at 4 days and 3–4 months postpartum were inversely related to infant birth weight (p = 0.006 and 0.03 respectively)
**XXI d: Widström et. al., 1991 [47]**	
	This relationship was only observed in non-smoking mothers
Maternal somatostatin levels and their correlation with infant birth weight	
(XXI a, XXI b, XX c and XXI d: articles based on the same material)	
	There was a positive correlation between infant birth weight and placental weight (p = 0.0001) and a negative correlation between somatostatin levels and placental weight (p = 0.04)
	In a regression model, somatostatin levels and smoking were shown to equally influence infant birthweight
	Levels of somatostatin during breastfeeding at 4 days postpartum were higher in smoking compared to non-smoking breastfeeding women (p<0.05 over several samples)
	There were no significant differences in milk yield between the two groups
	Smoking women had a shorter duration of exclusive breastfeeding vs. non-smoking women (p < 0.05)

(no abbreviations)

**Table 8:** Maternal personality and mood in connection with breastfeeding including women with medical interventions, during labour, birth or postpartum.

**WITHOUT MEDICAL INTERVENTIONS**	
**VI a: Cox et.al., 2015 [20]**	Oxytocin values at 8 weeks were significantly lower in women with high versus low depression and anxiety scores (p<0.05)
Oxytocin and HPA stress axis reactivity in postpartum women	The oxytocin AUC at 8 weeks was inversely correlated with scores for both depression (EPDS) and anxiety (STAI) scores (all groups p<0.01)
**VI b: Stuebe et. al., 2013 [34]** Association between maternal mood and oxytocin response to breastfeeding	
(VI a and VI b; articles based on the same material)	
**XI e: Jonas et.al., 2008 [51]**	Breastfeeding women at 2 days and 2 and 6 months after vaginal birth scored significantly lower in anxiety and aggression, and higher on socialization scales, compared to a normative group of non-pregnant, non-lactating women of similar age
Influence of oxytocin or epidural analgesia on personality profile in breastfeeding women: a comparative study	
	Scores were stable over time
(XI a, XI b, XI c, XI d and XI d; articles based on the same material)	
**XVIII b: Nissen et. al., 1998 [30]**	Breastfeeding women 2 days after vaginal birth scored lower on KSP somatic anxiety (p = 0.009), psychic anxiety (p = 0.001), muscular tension (p = 0.003), psychaesthenia (p = 0.0007), suspicion (p = 0.003), and guilt (p = 0.004), and higher scores in socialization (p = 0.002), compared to a normative group of non-pregnant, non-lactating women of similar age
Oxytocin, prolactin, milk production and their relationship with personality traits in women after vaginal delivery or Cesarean section	
	Basal oxytocin levels correlated with impulsiveness (p = 0.04)
	The number of oxytocin pulses correlated positively with social desirability (p = 0.009) and negatively with detachment (p = 0.05)
XVIII a and XVIII b; articles based on the same material)	
**XXI b: Uvnäs Moberg et.al., 1990 b [36]**	Breastfeeding women 4 days after vaginal birth scored lower in KSP muscular tension (p<0.05), monotony avoidance (p<0.001) and psychaesthenia (p<0.01) and higher in social desirability (p<0.01), compared to a normative group of non-pregnant, non-lactating women of similar age
Personality traits in women 4 days postpartum and their correlation with plasma levels of oxytocin and prolactin	
	Average oxytocin levels were negatively correlated with somatic anxiety, psychic anxiety, muscular tension and guilt (p<0.01 for all)
(XXI a, XXI b, XXI c and XXI d: articles based on the same material)	
	Average oxytocin levels were positively correlated with socialisation and social desirability (p<0.01)
**WITH MEDICAL INTERVENTIONS**	
**Emergency caesarean section**	
**XVIII b: Nissen et. al., 1998 [30]**	Breastfeeding women 2 days after caesarean section scored differently on KSP compared to vaginal birth
Oxytocin, prolactin, milk production and their relationship with personality traits in women after vaginal delivery or Cesarean section	Following caesarean, KSP was closer to normative than vaginal birth group, but lower than normative for psychic anxiety (p = 0.04) and psychaesthenia (p = 0.01)
	Compared with vaginal birth, women after caesarean scored higher for somatic anxiety (p = 0.08) muscular tension (p = 0.004) and suspicion (p = 0.09), and lower for social desirability (p = 0.007)
	Basal oxytocin levels were inversely correlated with psychic anxiety (p = 0.03) somatic anxiety (p = 0.02), psychaesthenia (p = 0.05) and suspicion (p = 0.04)
(XVIII a and XVIII b; articles based on the same material)	
	For the whole group, the number of oxytocin pulses during breastfeeding positively correlated with milk yield (p = 0.03), which correlated with social desirability (p = 0.03)
**Mixed interventions**	
**XI e: Jonas et. al,. 2008 [51]**	Breastfeeding women 2 days, and 2 and 6 months after birth scored differently on KSP according to labour interventions, compared to a normative group of non-pregnant, non-lactating women of similar age
Influence of oxytocin or epidural analgesia on personality profile in breastfeeding women: a comparative study	
	Women exposed to SOT IV in labour or SOT IM postpartum scored significantly lower in anxiety and aggression, and higher on socialization scales, compared to normative, with scores stable over time
	Among women exposed to EDA without SOT IV, scores were not different to normative at 2 days
(XI a, XI b, XI c, XI d and XI d; articles based on the same material)	However, at 2 and 6 months, EDA scores approached those of the other groups
	SOT IV, alone or together with EDA, was associated with decreased scores in inhibition of aggression (p = 0.019 at 2 days), psychaesthenia (p = 0.028 at 2 months) and muscular tension (p = 0.05 at 6 months)
	In the whole group, SOT IV dosage was negatively correlated with inhibition of aggression
**XXII: Velandia, 2012 [39]**	Breastfeeding women 2 days after prelabour caesarean section had similar KSP values to the normative group
Parent-infant skin-to-skin contact studies: Parent-infant interaction and oxytocin levels during skin-to-skin contact after cesarean section and mother-infant skin-to-skin contact as treatment for breastfeeding	Mothers with skin-to-skin contact after birth, and who were also administered additional SOT IV postpartum for medical reasons, have KSP restored to values normally observed in women after birth

AUC = area under the curve, IV = intravenous, IM = intramuscular EDA = epidural analgesia, SOT = synthetic oxytocin, EPDS = Edinburgh Postnatal Depression Scale, KSP = Karolinska Scales of Personality, STAI = State-Trait Anxiety Inventory, vs. = versus

### Measurement of oxytocin levels

The technique used for measurement of oxytocin levels is of critical importance for the interpretation of data. Radioimmunoassay or RIA is the gold standard for determination of oxytocin levels, whereas some techniques such as Enzyme Linked Immunoassay or ELISA, especially if performed without extraction of the samples, may give rise to 10–100 fold higher values, than those obtained by RIA. This is probably due to the fact that ELISA is a less specific method and may include measurement of fragments or metabolites of oxytocin or even of other material that is not related to the oxytocin molecule. Therefore values obtained with ELISA have to be interpreted with caution [48, 49].

Another important variable for the oxytocin levels obtained is of course the number of and timing of samples collected. As oxytocin is released in short lasting pulses in response to breastfeeding, repeated samples collected at short time intervals are needed in order to demonstrate the pattern of oxytocin release in response to breastfeeding [49]. As oxytocin is released almost immediately after the onset of breastfeeding, samples must be collected soon after initiation of breastfeeding in order to record the release. Also, quick blood sampling is more likely to catch the oxytocin induced peaks than is slow blood sampling because the peaks may become blunted, if blood samples are drawn slowly. A much more detailed discussion of the techniques used for determination of oxytocin levels in plasma as well as on the importance of timing of blood sampling was presented in a previously published paper [1]. Oxytocin levels in saliva have not been convincingly proven to reflect circulating oxytocin levels in connection with breastfeeding and have therefore been excluded from this study [49, 50].

## Results

### Effects of breastfeeding and other types of breast stimulation on oxytocin levels (3a)

#### Effects of breastfeeding and skin to skin contact

Basal levels of oxytocin decreased during the first days postpartum [37]. Maternal oxytocin levels were shown to rise in connection with the onset of suckling or other types of breast stimulation or skin-to-skin contact immediately after birth [14–42]. In control studies performed without suckling, or in studies of women formula-feeding their infants, no rise of oxytocin was observed [18, 20, 23].

#### Pattern of oxytocin release

A pulsatile pattern of oxytocin release was found in response to breastfeeding in particular during the first days after birth [23, 24, 28, 30, 32, 35, 37, 40]. When samples were collected frequently (every 30^th^ second), up to five short-lasting pulses were observed during the first 10 minutes of breastfeeding [24, 32, 35, 40]. Later on, during lactation, these peaks often coalesced, showing a larger and more protracted rise. Levels generally returned to baseline after 20 minutes of suckling. A second peak of oxytocin was sometimes observed, possibly in connection with suckling of the second breast [37].

Skin-to-skin contact immediately after birth gave rise to an increased oxytocin levels. No short-lasting peaks were observed, rather a more protracted type of oxytocin release [27, 31, 39].

#### Levels of oxytocin

Basal levels of oxytocin varied between 0 and 20 pg/mL, when measured with RIA. Oxytocin levels on average rose 5-fold (range 2-10-fold) in response to breastfeeding. The difference in the size of the peaks is due to variation between individual mothers, early versus late breastfeeding, but is also due to methodological reasons. The different assays used for oxytocin determination do not give rise to identical values and furthermore the timing of and, in fact also the duration of blood sampling, influences the size of the peaks. In the experiments in which oxytocin levels were measured with ELISA onset levels were high (132- to 1642 pg/mL) and even then peaks of oxytocin were induced by breastfeeding [14, 51].

In some studies, where mothers and babies were together before start of suckling, peaks of oxytocin were observed even before the onset of suckling, often in response to the baby’s cry [28].

As mentioned above, the magnitude of the rise of oxytocin increased over the duration of lactation [23, 37]. However, the length of suckling during a breastfeed episode did not correlate with oxytocin levels, or with the amount of oxytocin (area under the curve) released in connection with that particular breastfeed. There was, however a strong correlation between the amount of oxytocin released by the individual women at different times in lactation [37].

Oxytocin levels in response to breastfeeding were higher in multiparous than in primiparous women [26].

Basal oxytocin levels were higher in exclusively, compared to partially, breastfeeding women [23, 37], and fell after weaning [37].

#### Effects of breast massage

Manual breast massage was associated with a substantial and sustained elevation in oxytocin, without the pulses that occurred during suckling. In fact, more oxytocin was released by breast massage than by suckling [40].

#### Effects of stress, depression and alcohol

Women exposed to different types of stress (mental or noise) during breastfeeding had significantly fewer oxytocin peaks in response to breastfeeding than those mothers that were not exposed to a stressor [35]. Mothers with high depression scores had lower oxytocin levels, both before and during breastfeeding [20, 34]. Alcohol consumption (0.4 g/kg, a high amount) prior to breastfeeding also reduced oxytocin release [29].

#### Oxytocin and breastfeeding outcomes

In several of the studies, milk yield was measured [22, 26, 28–30, 32, 35, 37]. The number of breastfeeding-induced oxytocin pulses during early breastfeeding correlated with milk yield during suckling [30], and with the subsequent duration of lactation [32]. In some studies, a correlation between oxytocin levels and milk yield was found [26, 29]. Higher birth weight of the infant correlated with higher oxytocin levels during suckling at 3–4 months [37].

#### Effect of mechanical breast stimulation

Mechanical breast pumping was followed by a rise of oxytocin levels, which was generally of similar amplitude to the release caused by suckling [41]. However, no oxytocin peaks were observed before the onset of mechanical pumping [25]. More oxytocin was released in response to double pumping (both breasts together) compared to single pumping. In addition double pumping produced the highest milk yield, indicating a relationship between oxytocin levels and milk yield [17, 41].

In healthy, late-pregnant (non-breastfeeding) women, mechanical pumping or nipple stimulation caused a release of oxytocin, which was associated with uterine contractions [15, 19]. Oxytocin levels also increased in response to breast stimulation in healthy non-pregnant, non-breastfeeding women [15, 18].

### Oxytocin levels with medical interventions (Table 4)

In five of the included articles, data on the effects of medical interventions on oxytocin levels during breastfeeding were reported [24, 30, 32, 39, 42].

Medical interventions in connection with birth influenced the release of oxytocin in response to skin-to-skin contact and suckling. Women who had had a prelabour caesarean section did not release oxytocin in response to skin-to-skin contact and suckling after birth [39]. In women who received a postpartal intravenous infusion of synthetic oxytocin to prevent bleeding in the postpartum period, these effects were significant, i.e. oxytocin was released in response to skin-to-skin contact and suckling [39].

Women, who had had a caesarean section, had significantly fewer pulses of oxytocin during a breastfeeding episode on day two after birth [32], which was correlated with reduced milk yield and reduced subsequent duration of lactation [30].

Women who had received both epidural analgesia and an intravenous infusion of synthetic oxytocin during labour had lower oxytocin levels in connection with breastfeeding two days after birth, compared to those with either intervention alone, or to unexposed women [24]. This decrease was dose-dependent; the more oxytocin and epidural analgesia they had received, the lower their oxytocin levels in response to breastfeeding at two days postpartum [24]. These data must, however, be interpreted with cautions as they were measured with ELISA, which is a less specific method, and which gives rise to higher basal levels of oxytocin than RIA.

### Prolactin levels (Table 5)

#### Effect of breastfeeding

Prolactin levels were measured in 16 of 29 studies [14, 15, 20, 21, 23–25, 28–30, 32, 35, 37, 38, 40, 41]. Prolactin levels rose in response to breastfeeding in all 15 studies. The rise of prolactin in response was gradual and generally not significant until 10 minutes after suckling commencement. Sustained prolactin elevations persisted for at least 60 minutes [21, 23–25, 28, 32, 35, 37, 38, 40].

No rise of prolactin was observed in the absence of breastfeeding or prior to breastfeeding [24, 28]. Nor did prolactin levels increase with nipple stimulation or breast tactile massage [15, 40]. Stress (mental and noise) during breastfeeding did not influence breastfeeding-induced prolactin release levels [33].

There was a significant positive correlation between the duration of the breastfeeding episode and median prolactin levels during these episodes [24, 30]. In addition there was a clear correlation between basal oxytocin and prolactin levels [30], and between oxytocin variability (reflecting pulsatility) and the rise in prolactin in response to suckling [24]. Prolactin levels, both basal and in response to suckling decreased over the course of lactation [23, 37].

#### Prolactin and breastfeeding outcomes

No correlation was found between suckling-induced prolactin levels and the amount of milk produced during a particular breastfeeding session [28, 35, 37]. In one study, prolactin levels at 3–4 months predicted the remaining duration of exclusive breastfeeding [37]. This could reflect the amount and intensity of suckling performed by the breastfeeding infant during previous breastfeeding sessions.

#### Effect of mechanical breast stimulation

Prolactin levels increased in response to mechanical breast pumping, reaching similar levels as during breastfeeding [17, 25, 41]. When two pumps were applied at the same time, prolactin levels were higher compared to single pumping [17, 41] and milk yield was greater [41].

#### Effect of medical interventions

The rise of prolactin levels seen in response to breastfeeding at two days postpartum was significantly lower in women who had had an emergency [24] caesarean section, compared to women who had had a vaginal birth [32].

Breastfeeding at two days postpartum did not induce any significant rise of prolactin levels in women who had received epidural analgesia during labour [24]. In contrast, women who had received an intravenous infusion of synthetic oxytocin during labour, had significantly higher suckling-induced prolactin levels, compared to women, who had not had any interventions. [24].

### Stress variables (Table 6)

Stress variables were measured in nine of the included articles/references [14, 16–18, 20, 30, 32, 34, 42]. The data are reported in 12 articles, since for one of the included studies [24] breastfeeding induced effects on stress levels were reported in 3 additional separate articles [43–45].

#### Effect of breastfeeding

Suckling was seen to induce a significant decrease in ACTH (adrenocorticotrophic hormone, which stimulates the release of cortisol from the adrenal cortex*)* [18, 43]. Suckling also induced a significant decrease in cortisol levels [16–18, 20, 32, 43].

The decrease in ACTH levels was positively correlated to the duration of suckling during a breastfeeding episode [43] and negatively correlated to both oxytocin levels and oxytocin variability [18, 43].

The decrease in cortisol levels during suckling was significantly correlated with the decrease of ACTH levels, but not with the duration of the breastfeeding episode. In contrast, the decrease in cortisol levels was significantly correlated to the duration of skin-to-skin contact prior to breastfeeding [43].

No decrease of ACTH or cortisol levels was observed in control experiments without breastfeeding, or after formula-feeding [16, 18].

Both systolic and diastolic blood pressure decreased in response to breastfeeding [32, 44, 45]. Basal blood pressure was significantly reduced in women after 6 weeks of breastfeeding, but each breastfeeding session was still associated with a further decrease in blood pressure [45].

Breastfeeding induced a long term stress buffering effect; after an episode of breastfeeding, physiological responses to external stressors, including heart rate and cortisol response, were reduced [20].

#### Effect of mechanical breast stimulation

Mechanical breast-pumping in lactating women decreased ACTH and cortisol levels. The nadir of ACTH levels coincided with the maximal rise in oxytocin levels [18]. A decrease of ACTH and cortisol levels was also observed in response to mechanical pumping in non-lactating normal cycling women, demonstrating that this effect is not restricted to lactating women. Again, the lowest levels of ACTH correlated in time with the highest oxytocin levels [18].

#### Effects of medical interventions

No significant differences were found in the decrease of cortisol levels during breastfeeding between vaginal delivery or emergency caesarean section [32].

Both basal and breastfeeding-related cortisol levels were lower in women who had experienced epidural labour analgesia compared to women who had received an intrapartum intravenous infusion of synthetic oxytocin, either alone or in combination with epidural analgesia [43].

Emergency caesarean section did not influence the fall in blood pressure caused by breastfeeding two days after birth [32]. In contrast, basal blood pressure in women who had received epidural analgesia during birth was significantly lower than in the other groups, and no decrease in blood pressure was observed in response to breastfeeding [44].

Mothers who had a prelabour caesarean section with immediate skin-to-skin contact and who had also received a postpartum intravenous infusion of synthetic oxytocin, showed a significant increase in antioxidant measures. The postpartum rise in oxytocin levels was correlated with these effects [42].

### Digestion, metabolism and nutrition (Table 7)

Measures of digestion, metabolism and nutrition were reported in three of the included articles [22, 46, 47]. The data are reported in five articles, since for one of the included studies [37] breastfeeding induced effects on somatostatin release were reported in two separate articles [46, 47].

#### Effect of breastfeeding

Somatostatin inhibits the activity of the endocrine system of the gastrointestinal tract, e.g the release of gastrin from the stomach, that stimulates gastric acid secretion and insulin and glucagon from the pancreas that, participate in the control of glucose levels [46, 47]. Levels of somatostatin tended to fall in response to suckling at 3–4 days after birth. In contrast, somatostatin levels consistently rose in response to suckling at 3–4 months postpartum [46]. Average somatostatin levels recorded in connection with breastfeeding at 3–4 months postpartum were significantly higher than those obtained at 3–4 days after birth [46]. A strong correlation was found between the release of somatostatin at 3–4 days and at 3–4 months postpartum [46].

The weight of the newborn correlated strongly and inversely with somatostatin levels obtained at 3–4 days and 3–4 months postpartum and also with placental weight [46].

Somatostatin levels were higher at 4 days in smoking compared to non-smoking women. Smoking was associated with shorter duration of breastfeeding [47].

Breastfeeding was also associated with a rise of the levels of thyroid stimulating hormone (TSH) [20].

Thirst was increased in response to breastfeeding, and the amount of water ingested after breastfeeding was associated with intensity of thirst reported [22].

### Personality traits and mood (Table 8)

Personality traits and mood were reported in five of the included articles [20, 30, 34, 36, 39, 51].

The data are reported in six articles, since for one of the included studies [24] data on personality traits are presented in a separate article [51].

#### Effect of breastfeeding

In four studies [30, 36, 39, 51] the scores of items related to anxiety, tension and aggression were decreased, and the scores of items related to social functioning were elevated, in comparison to a normative group of non-pregnant non-breastfeeding women of the same age. These changes persisted for 6 months, if the mothers continued to exclusively breastfeed [51].

In these studies, oxytocin levels during breastfeeding correlated negatively with KSP anxiety and tension scores, and positively with scores in social functioning [30, 36]. In other studies, women with higher scores on depression “Edinburgh Postnatal Depression Scale” (EPDS) and anxiety “State-Trait Anxiety Inventory” (STAI) had lower oxytocin release during breastfeeding at 8 weeks [20, 34].

#### Effect of medical interventions

Mothers who had given birth by pre-labour or emergency caesarean section did not have the full pattern of KSP maternal adaptations compared to women who had a vaginal birth [30, 39]. In women who received epidural analgesia (without infusion of synthetic oxytocin), no maternal adaptations on KSP were found at eight weeks. However, adaptations were restored at two-six months in women who exclusively breastfed [24].

Infusions of synthetic oxytocin during labour slightly enhanced the KSP adaptations observed in connection with breastfeeding two days postpartum. The dosage of synthetic oxytocin administered was negatively correlated with the scores of inhibition of aggression [39, 51]. In addition, infusions of synthetic oxytocin restored the lack of the changes in the KSP seen in mothers who had received epidural analgesia alone. Finally, oxytocin infusions given postpartum restored the lack of changes in the KSP pattern found in women who had had a prelabour caesarean section [51].

## Discussion

Articles in which blood/plasma levels of oxytocin levels in response to breastfeeding had been recorded were included in this systematic literature review. In some of the selected articles (and in some of the associated articles based on the same clinical studies) information was also reported regarding prolactin, stress, nutrition, metabolism, as well as personality and mood. Some studies also reported on effects of medical interventions in connection with birth. Since the primary objective of this systematic literature review was to summarize studies that measured breastfeeding-related oxytocin levels, we obviously did not identify all studies regarding the secondary variables mentioned above. We chose to include the secondary oxytocin linked effects because data obtained within the same article may reveal more causal with oxytocin relationships than data obtained from different articles. In a sense this gives a deeper meaning to the oxytocin levels recorded because they also become a proxy for the secondary oxytocin linked endocrine, physiological and psychological effects.

The studies included in this review differ in many ways. Some studies were performed during early breastfeeding and some later on during established breastfeeding. In some studies oxytocin release in response to suckling was studied immediately after birth. The timing of blood samples was also very different. The number of blood samples collected in the different studies varied between 2 and 24 and the frequency by which samples were collected between 30 seconds and 20 minutes. In other studies, as much as 24 blood samples were collected, some of them with 30 second intervals. We have tried to describe and summarize the results regarding breastfeeding induced effects on oxytocin level despite these methodological differences.

All of the studies showed that suckling is associated with oxytocin release [14–42]. In early breastfeeding, the release of oxytocin occurred in pulses, which later on during breastfeeding coalesced into a large sustained peak [23, 24, 28, 30, 32, 35, 37, 40]. There were correlations between oxytocin levels and milk yield in several of the included studies and also correlations with duration of breastfeeding and weight of the newborn [14, 22, 25, 26, 28–30, 32, 35–37, 52]. The correlation between milk yield and the number of oxytocin pulses is of particular interest as each peak of oxytocin is linked to milk ejection [53]. Taken together, these data suggest that oxytocin levels in response to breast feeding correlates with milk production and also for the duration of breastfeeding.

From this perspective, it is of interest that the number of oxytocin peaks released during breastfeeding was reduced by relatively minor stressors, such as noise and mental activity (concentration), indicating that both an “external” and “internal” environment characterized by calmness are of importance for the breastfeeding mother in order promote the milk-ejection reflex and thereby milk production [35]. Also maternal mood influenced oxytocin levels, as depression was associated with lower oxytocin release in response to breastfeeding [34].

Oxytocin produced in the supraoptic (SON) and paraventricular nuclei (PVN) of the hypothalamus is released from the posterior pituitary into the circulation in response to breastfeeding in order to stimulate milk ejection. However, oxytocin is at the same time released from oxytocinergic nerves that project from the PVN in the hypothalamus to many regulatory areas in the brain. In this way breastfeeding may be accompanied by many different physiological and psychological adaptations, which help mothers to cope with the demands of breastfeeding and motherhood. For a more elaborate description of the central effects of oxytocin and how oxytocin release into the circulation and the brain are coordinated, see Uvnäs Moberg et. al. 2019 [1].

**Fig 2:**
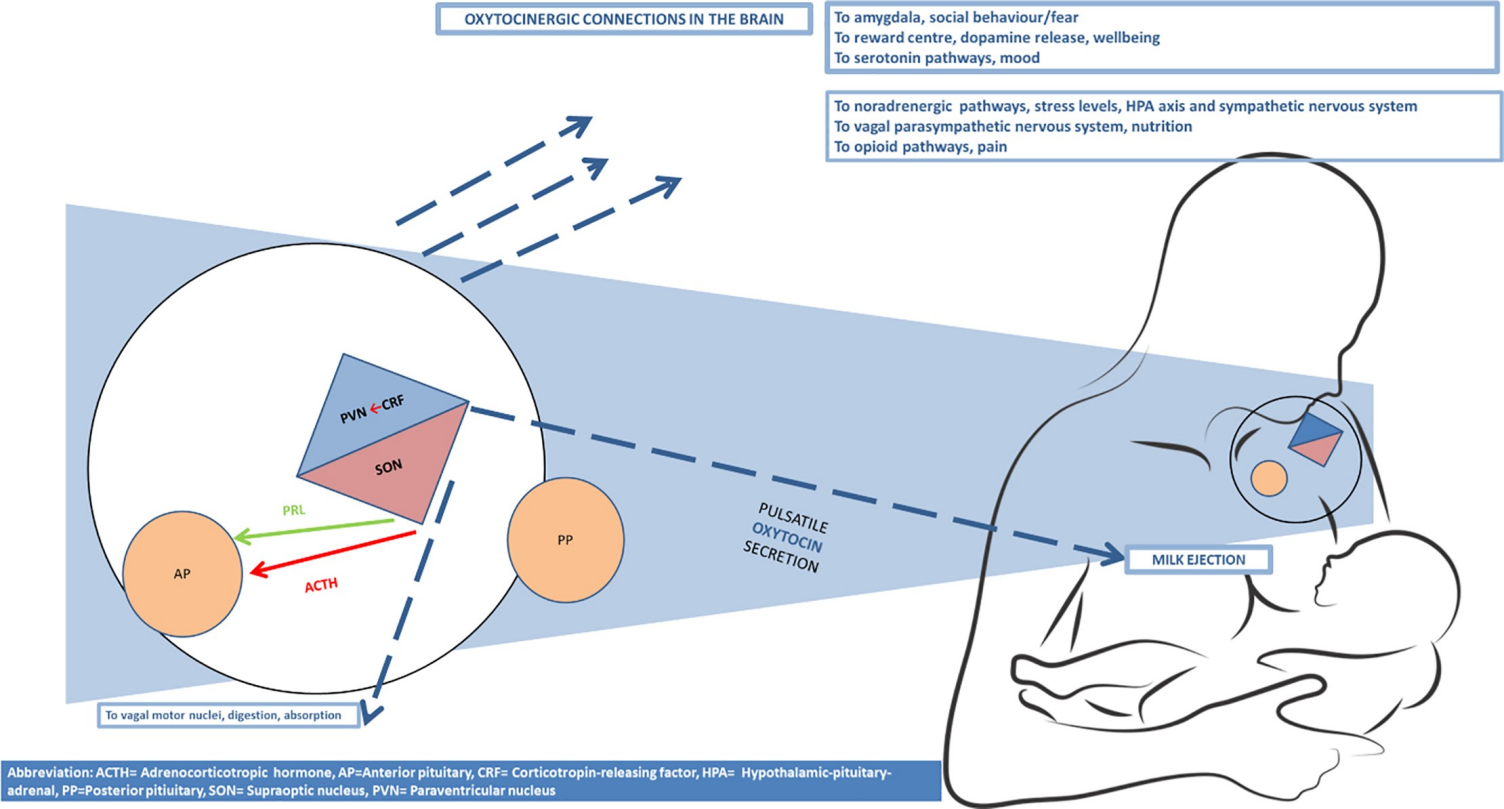
Schematic illustration of the oxytocin pathways activated during breastfeeding.

The present results identified multiple adaptive effects of breastfeeding, which are linked to activation of the oxytocin system. These effects include facilitation of prolactin release, decreased activity in the stress system (HPA axis: ACTH and cortisol and the sympathetic nervous system: blood pressure and heart rate) and increased function of the parasympathetic part of the autonomic nervous system, including activation of the gastrointestinal tract by the vagal nerve. Oxytocin release was also correlated with mental adaptations that facilitate motherhood such as increased levels of social interaction and decreased anxiety [1]. The oxytocinergic pathways in the brain that may be involved in these actions are illustrated in Fig 2.

As reported in this review prolactin was released by suckling [15, 20, 21, 23–25, 28–30, 32, 35, 37, 38, 40, 41]. The release of prolactin, however, differed from oxytocin release caused by suckling in many ways. During a breastfeeding episode, oxytocin is released within minutes and in a pulsatile pattern, whereas prolactin levels increase more slowly, are not pulsatile and more long lasting than oxytocin levels. Nor could prolactin be released by breast-massage and prolactin release was less sensitive to acute stressors than oxytocin release. Altogether, these data indicate that oxytocin and prolactin release are in part mediated by separate mechanisms and that prolactin release is more resilient to stress than is oxytocin [35]. Nevertheless, oxytocin exerts an over-riding facilitating influence on prolactin release, via the oxytocin-containing nerves that extend from the PVN to the prolactin-producing cells in the anterior pituitary, as shown above and in Fig 2. In this way, oxytocin stimulates milk production as well as milk ejection [7].

Breastfeeding was also linked to anti-stress effects, with decreases in ACTH, cortisol and blood pressure during a breastfeeding episode [16–18, 20, 30, 32, 34, 42–44, 51]. The decrease in ACTH levels was linked to the duration of suckling reflecting the inhibitory effect of oxytocin released within the PVN on Corticotropin-releasing factor (CRF), which regulates ACTH release. In addition, oxytocin may have a direct inhibitory effect on ACTH via nerves linking the PVN to ACTH-producing cells in the anterior pituitary (Fig 2).

In these studies, the decrease in cortisol levels was not only associated with reduced ACTH levels, but also to the duration of skin-to-skin contact. This latter effect most likely reflects the influence of oxytocin nerves that project to areas in the brain that regulate the function of the autonomic nervous system, and which are activated in response to stimulation of sensory nerves that originate in the skin. In the same way the decrease in blood pressure induced by suckling involves an oxytocin mediated decrease of the activity in the sympathetic nervous system [7, 49].

Lowering of ACTH and cortisol levels was also induced in response to suckling in non-breastfeeding women, suggesting that suckling may induce stress reduction in the absence of breastfeeding [18].

One of the studies showed that the sensitivity to stress is decreased for some hours following breastfeeding, indicating that breastfeeding is, in addition to the acute anti stress effect, linked to a more long-term stress-buffering effect [20]. Similar findings have been reported in other studies [54]. Taken together these data show that mothers are subject to powerful anti-stress effects during suckling, which may help them relax and adapt to breastfeeding. The antioxidant effects observed in response to skin-to-skin contact represent yet another “health” promoting effect of breastfeeding [44].

The data in this review also showed that thirst is increased and that, absorption, digestion and metabolism of nutrients were optimized by breastfeeding-related oxytocin release [22, 46, 47]. These effects assist in adapting the mother to the high demands of nutrition and fluid intake during breastfeeding. Oxytocin influences the gastrointestinal function by increasing the function of the vagal nerve. For example, it may decrease the release somatostatin, a hormone produced in the gastrointestinal tract, which exerts inhibitory effects on gastrointestinal function [7]. Oxytocin (and breastfeeding) lower the levels of somatostatin and thereby the function of the gastrointestinal tract increases. The finding of an inverse relationship between levels of oxytocin and somatostatin (which inhibits the activity of the endocrine system of the gastrointestinal tract) also supports the important role oxytocin has in adapting maternal metabolism to the demands of breastfeeding [55].

In several of the included studies, the personality inventory, Karolinska Scales of Personality (KSP) was applied to breastfeeding women [30, 36, 39, 51]. The KSP has 135 items in a four-point response format. It consists of 15 self-reported scales that can be divided into the anxiety proneness scales, the extroversion related scales, the socialization scale, the social desirability scale and the aggression-hostility related scales. The KSP is standardized for sex and age and the normative sample is based on 200 non-pregnant or lactating randomly selected women. The test has a high validity and retest stability [56, 57].

It seems that oxytocin release during breastfeeding influences maternal psychology in a way that facilitates motherhood, i.e. levels of anxiety and aggression are decreased, and social functioning increased. Breastfeeding is also linked to increased sensitivity in the mother and other mental adaptations that facilitate motherhood [6]. In a study performed by Strathearn et. al., 2009, dopamine activity in the maternal brain was increased in response to their own smiling baby’s photo. This increase in dopamine activity was related to maternal oxytocin levels and also to secure attachment [58].

These changes, which are linked to oxytocin levels are beneficial for the mother and might constitute a slight reflection of the more developed maternal behaviours that occur in other mammals in response to central oxytocin release, during parturition and suckling [59]. Altogether, these data indicate that the oxytocin release associated with breastfeeding contributes to the development of positive maternal experiences, psychological skills and physiological adaptations.

Mechanical breast stimulation was associated with oxytocin release and milk yield of similar magnitude as breastfeeding. Mechanical breast stimulation also stimulated prolactin release and decreased ACTH and cortisol levels [17, 25, 41]. These data suggest that mechanical breast stimulation is associated with at least some of the oxytocin-induced adaptations seen in response to breastfeeding [7].

By contrast, none of these adaptations were induced when mothers were bottle-feeding their babies [23]. Also, other studies have demonstrated that bottle feeding mothers are less sensitive in the communication with their babies and that they have higher stress levels than breastfeeding mothers [60].

Medical interventions during labour and birth have been associated with disturbed breastfeeding [8–10]. Such effects might potentially be mediated by a disrupted function of the oxytocin system during labour and birth, which then persists during breastfeeding. During labour the release of oxytocin is triggered by pressure from the baby’s head on the cervix and the vagina in response to uterine contractions (the Ferguson reflex). Less oxytocin may be released during caesarean sections (especially by prelabour caesarean sections due to the absence of uterine contractions and labour) than during vaginal birth [1]. Also, epidural analgesia may reduce oxytocin release in connection with birth. Epidural analgesia blocks the transmission in pain fibres, but also the transmission in the nerve fibres mediating the Ferguson reflex and thereby oxytocin release [61]. It is biologically plausible that such effects on oxytocin release induced by medical interventions in connection with birth could become long-lasting, with consequences for breastfeeding outcomes and other oxytocin-linked effects [1, 62]. In fact, results from some of the included studies indicate that oxytocin release in response to breastfeeding may be deranged by medical interventions. In women who had an emergency cesarean section, oxytocin release in response to early breastfeeding was significantly reduced [30, 32]. Prolactin release and maternal adaptations were also reduced, possibly as a consequence of the reduced release of oxytocin in the brain during breastfeeding [30, 32]. In another study mothers who had had a prelabour caesarean section, skin-to-skin contact immediately after birth did not stimulate maternal oxytocin release or maternal adaptations as measured with the KSP 2 days later [39]. Epidural analgesia was linked to decreased prolactin levels and decreased maternal adaptations [24, 51]. Taken together these data suggest that mothers who have given birth by caesarean or have had an epidural analgesia might at least initially during breastfeeding have a compromised function of the oxytocin system. This may contribute to the breastfeeding difficulties that women can experience following caesarean section and epidural analgesia [8, 9].

Both prolactin release, and maternal mental adaptations were, however, more strongly developed in women who received synthetic oxytocin infusions during labour, in comparison to those who did not receive any medical interventions. Some of these effects were dose-dependent [24, 51]. In addition, infusions of synthetic oxytocin seemed to counteract the negative influence of epidural analgesia on maternal mental adaptations [51]. Infusions of synthetic oxytocin also restored the release of endogenous oxytocin in response to early skin-to-skin contact, as well as the development of maternal mental adaptations, which was otherwise not developed following prelabour caesarean section [39]. These central effects of synthetic oxytocin are probably induced indirectly via nervous reflexes, such as reinforcement of the Ferguson reflex (1).

In addition, the repetitive release of endogenous oxytocin during breastfeeding may counteract some of the negative effects of caesarean section and epidural analgesia on prolactin release and maternal mental adaptations [51]. In this way breastfeeding may counteract some of the negative consequences caused by medical interventions that are linked to reduction of oxytocin release in labour and birth.

The repetitive release of oxytocin occurring during breastfeeding may also explain why basal blood pressure was significantly decreased after 6 weeks of breastfeeding [45]. Such long term effects of repeated oxytocin exposure may also explain why breastfeeding is linked to long term health-promoting effects [63]. In particular, there are long term benefits for different types of cardiovascular disease, including hypertension, stroke, heart infarction and diabetes type 2, with greater effects for longer duration of lactation [4, 5, 64–66].

Also the infants profit from breastfeeding beyond being exposed to the beneficial components of breastmilk [67]. Skin-to-skin contact and suckling promote oxytocin release also in the baby [7], with possible long term beneficial effects on bonding, health and wellbeing [4, 6, 7].

Taken together this review shows that breastfeeding is more than a transfer of breastmilk from mother to infant. By stimulating the release of oxytocin into the circulation and into the brain, it not only stimulates milk ejection, it also stimulates neuroendocrine processes that facilitate milk production and maternal physiological and psychological adaptations. Also, the baby is exposed to oxytocin during breastfeeding with possible long-term beneficial effects on health and wellbeing. When considering these positive aspects of breastfeeding it is obvious that breastfeeding should be valued beyond its role as a source of breastmilk for the baby.

## Conclusions and implications for clinical practice

The Baby-Friendly Hospital Initiative (BFHI) which is supported by the WHO focuses on breastfeeding for healthy, mother-infant dyads. To stimulate and facilitate breastfeeding “The ten steps to successful breastfeeding” have been formulated. They recommend immediate and uninterrupted skin-to-skin contact and support of mothers to initiate breastfeeding as soon as possible after birth. They also support mothers to initiate and maintain breastfeeding and how to manage common breastfeeding difficulties. Further, they support the mothers and their infants to remain together and to practise rooming in the maternity ward and not to provide breastfed newborns any food or fluids other than breast milk, unless medically indicated. Finally, they also provide support for mothers to recognize and respond to their infants’ cues for feeding. There is substantial evidence that implementing the “Ten Steps” significantly improves breastfeeding rates. A systematic review of 58 studies on maternity and newborn care published in 2016 demonstrated clearly that adherence to the Ten Steps impacts early initiation of breastfeeding immediately after birth, exclusive breastfeeding, and total duration of breastfeeding [68].

In fact, all the practical recommendations described within “The ten steps” are consistent with an optimized stimulation of oxytocin release. As summarized in this review oxytocin is released in response to skin-to-skin contact and breastfeeding to cause milk ejection and to promote milk production. It also induces physiological changes and psychological adaptations to facilitate motherhood. The relevance of the “Ten steps” recommendations is therefore supported by their link to activation of the oxytocin system. More research should be performed to increase the knowledge about the link between the practical recommendations mention above in BFHI and the activation of the oxytocin system in mothers and their babies.

Repeated exposure to oxytocin with each episode of breastfeeding, may contribute to the lifelong benefits of breastfeeding for mother and baby and it may even counteract some negative consequences of medical interventions.

It is of importance that health professionals receive information about how oxytocin and oxytocin linked effects promote breastfeeding and how this system is reinforced by the recommendations of BFHI.

## Supporting information

S1 Checklist PRISMA 2009 checklist. (DOC)

S1 File Search strategy. (DOCX)

## References

[1] Uvnäs-Moberg K, Ekström-Bergström A, Berg M, Buckley S, Pajalic Z, Hadjigeorgiou E, et al Maternal plasma levels of oxytocin during physiological childbirth–a systematic review with implications for uterine contractions and central actions of oxytocin. BMC Pregnancy and Childbirth. 2019;19(285). 10.1186/s12884-019-2365-9 PMC6688382 31399062

[2] World Health Organization. Breastfeeding 2017. Available from: http://www.who.int/maternal_child_adolescent/topics/newborn/nutrition/breastfeeding/en/.

[3] UNICEF. Breastfeeding 2013. Available from: https://www.unicef.org/nutrition/index_breastfeeding-ten-steps.html.

[4] Victora CG, Bahl R, Barros AJ, França GV, Horton S, Krasevec J, et al Breastfeeding in the 21st century: epidemiology, mechanisms, and lifelong effect. The Lancet. 2016;387(10017):475–90. 10.1016/S0140-6736(15)01024-7 26869575

[5] Nguyen B, Gale J, Nassar N, Bauman A, Joshy G, Ding D. Breastfeeding and Cardiovascular Disease Hospitalization and Mortality in Parous Women: Evidence From a Large Australian Cohort Study. Journal of the American Heart Association. 2019;8(6):p.e011056 10.1161/JAHA.118.011056 30871389 PMC6475066

[6] Tharner A, Luijk MP, Raat H, IJzendoorn MH, Bakermans-Kranenburg MJ, Moll HA, et al Breastfeeding and its relation to maternal sensitivity and infant attachment. Journal of Developmental & Behavioral Pediatrics. 2012;33(5):396–404. 10.1097/DBP.0b013e318257fac3 22580735

[7] Uvnäs-Moberg K. Oxytocin: The Biological Guide to Motherhood: Praeclarus Press, LLC; 2014.

[8] Prior E, Santhakumaran S, Gale C, Philipps LH, Modi N, Hyde MJ. Breastfeeding after cesarean delivery: a systematic review and meta-analysis of world literature. The American journal of clinical nutrition. 2012;95(5):1113–35. 10.3945/ajcn.111.030254 22456657

[9] French CA, Cong X, Chung K, Sam. Labor epidural analgesia and breastfeeding: a systematic review. Journal of Human Lactation. 2016;32(3):507–20. 10.1177/0890334415623779 27121239

[10] Erickson EN, Emeis CL. Breastfeeding outcomes after oxytocin use during childbirth: an integrative review. Journal of midwifery women's health. 2017;62(4):397–417. 10.1111/jmwh.12601 28759177

[11] Liberati A, Altman DG, Tetzlaff J, Mulrow C, Gøtzsche PC, Ioannidis JP, et al The PRISMA statement for reporting systematic reviews and meta-analyses of studies that evaluate health care interventions: explanation and elaboration. PLoS medicine. 2009;6(7):e1000100 10.1371/journal.pmed.1000100 19621070 PMC2707010

[12] Covidence. A Cochrane technology platform—systematic review management 2019. Available from: www.covidence.org.

[13] Lara-Cinisomo S, McKenney K, Di Florio A, Meltzer-Brody S. Associations between postpartum depression, breastfeeding, and oxytocin levels in Latina mothers. Breastfeeding Medicine. 2017;12(7):436–42. 10.1089/bfm.2016.0213 28749705 PMC5646739

[14] Erickson EN, Carter CS, Emeis CL. Oxytocin, vasopressin and prolactin in new breastfeeding mothers: Relationship to clinical characteristics and infant weight loss. Journal of Human Lactation. 2019. doi: 0890334419838225. 10.1177/0890334419838225 PMC9766886 31033381

[15] Amico JA, Finley B. Breast stimulation in cycling women, pregnant women and a woman with induced lactation: pattern of release of oxytocin, prolactin and luteinizing hormone. Clinical Endocrinology. 1986;25(2):97–106. 10.1111/j.1365-2265.1986.tb01670.x 3791664

[16] Amico JA, Johnston JM, Vagnucci AH. Suckling-induced attenuation of plasma cortisol concentrations in postpartum lactating women. Endocrine research. 1994;20(1):79–87. 10.3109/07435809409035858 8168464

[17] Chatterton RT Jr, Hill PD, Aldag JC, Hodges KR, Belknap SM, Zinaman MJ. Relation of plasma oxytocin and prolactin concentrations to milk production in mothers of preterm infants: influence of stress. The Journal of Clinical Endocrinology Metabolism. 2000;85(10):3661–8. 10.1210/jcem.85.10.6912 11061519

[18] Chiodera P, Salvarani C, Bacchi-Modena A, Spallanzani R, Cigarini C, Alboni A, et al Relationship between plasma profiles of oxytocin and adrenocorticotropic hormone during suckling or breast stimulation in women. Hormone Research in Paediatrics. 1991;35(3–4):119–23. 10.1159/000181886 1666892

[19] Christensson K, Nilsson BA, Stock S, Matthiesen AS, Uvnäs‐Moberg K. Effect of nipple stimulation on uterine activity and on plasma levels of oxytocin in full term, healthy, pregnant women. Acta obstetricia et gynecologica Scandinavica. 1989;68(3):205–10. 10.3109/00016348909020990 2618602

[20] Cox E, Stuebe A, Pearson B, Grewen K, Rubinow D, Meltzer-Brody S. Oxytocin and HPA stress axis reactivity in postpartum women. Psychoneuroendocrinology. 2015;55:164–72. 10.1016/j.psyneuen.2015.02.009 25768266 PMC4380745

[21] Dawood MY, Khan-Dawood FS, Wahi RS, Fuchs F. Oxytocin release and plasma anterior pituitary and gonadal hormones in women during lactation. The Journal of Clinical Endocrinology. 1981;52(4):678–83. 10.1210/jcem-52-4-678 6782115

[22] James R, Irons D, Holmes C, Charlton A, Drewett R, Baylis P. Thirst induced by a suckling episode during breast feeding and its relation with plasma vasopressin, oxytocin and osmoregulation. Clinical endocrinology. 1995;43(3):277–82. 10.1111/j.1365-2265.1995.tb02032.x 7586595

[23] Johnston JM, Amico JA. A prospective longitudinal study of the release of oxytocin and prolactin in response to infant suckling in long term lactation. The Journal of Clinical Endocrinology Metabolism. 1986;62(4):653–7. 10.1210/jcem-62-4-653 3949949

[24] Jonas J, L M, Nissen E, Ejdebäck M, Ransjö-Arvidson A, Uvnäs-Moberg K. Effects of intrapartum oxytocin administration and epidural analgesia on the concentration of plasma oxytocin and prolactin, in response to suckling during the second day postpartum. Breastfeeding Medicine. 2009;4(2):71–82. 10.1089/bfm.2008.0002 19210132

[25] Leake RD, Waters CB, Rubin RT, Buster JE, Fisher DA. Oxytocin and prolactin responses in long-term breast-feeding. Obstetrics gynecology. 1983;62(5):565–8. 6684741

[26] Lucas A, Drewett R, Mitchell M. Breast-feeding and plasma oxytocin concentrations. British Medical Journal,. 1980;281(6244):834–5. 10.1136/bmj.281.6244.834 7191754 PMC1714246

[27] Matthiesen AS, Ransjö‐Arvidson AB, Nissen E, Uvnäs‐Moberg K. Postpartum maternal oxytocin release by newborns: effects of infant hand massage and sucking. Birth. 2001;28(1):13–9. 10.1046/j.1523-536x.2001.00013.x 11264623

[28] McNeilly AS, Robinson I, Houston MJ, Howie PW. Release of oxytocin and prolactin in response to suckling. Br Med J. 1983;286(6361):257–9. 10.1136/bmj.286.6361.257 6402061 PMC1546473

[29] Mennella JA, Pepino MY, Teff KL. Acute alcohol consumption disrupts the hormonal milieu of lactating women. The Journal of Clinical Endocrinology Metabolism. 2005;90(4):1979–85. 10.1210/jc.2004-1593 15623810 PMC1351273

[30] Nissen E, Gustavsson P, Widström A, Uvnäs-Moberg K. Oxytocin, prolactin, milk production and their relationship with personality traits in women after vaginal delivery or Cesarean section. Journal of Psychosomatic Obstetrics Gynecology. 1998;19(1):49–58. 10.3109/01674829809044221 9575469

[31] Nissen E, Lilja G, Widström AM, Uvnás‐Moberg K. Elevation of oxytocin levels early post partum in women. Acta obstetricia et gynecologica Scandinavica. 1995;74(7):530–3. 10.3109/00016349509024384 7618451

[32] Nissen E, Uvnäs-Moberg K, Svensson K, Stock S, Widström A-M, Winberg J. Different patterns of oxytocin, prolactin but not cortisol release during breastfeeding in women delivered by caesarean section or by the vaginal route. Early Human Development. 1996;45(1–2):103–18. 10.1016/0378-3782(96)01725-2 8842644

[33] Piron-Bossuyt C, Bossuyt A, Vanden RD. Plasma oxytocin levels during lactation (author's transl). Annales d'endocrinologie. 1978;39(2):155–6. 686656

[34] Stuebe AM, Grewen K, Meltzer-Brody S. Association between maternal mood and oxytocin response to breastfeeding. Journal of women's health. 2013;22(4):352–61. 10.1089/jwh.2012.3768 23586800 PMC3627433

[35] Ueda T, Yokoyama Y, Irahara M, Aono T. Influence of psychological stress on suckling-induced pulsatile oxytocin release. Obstetrics and gynecology. 1994;84(2):259–62. 8041543

[36] Uvnäs-Moberg K, Widström A-M, Nissen E, Björvell H. Personality traits in women 4 days postpartum and their correlation with plasma levels of oxytocin and prolactin. Journal of Psychosomatic Obstetrics Gynecology. 1990;11(4):261–73. 10.3109/01674829009084422

[37] Uvnäs‐Moberg K, Widström AM, Werner S, Matthiesen AS, Winberg J. Oxytocin and prolactin levels in breast‐feeding women. Correlation with milk yield and duration of breast‐feeding. Acta obstetricia et gynecologica Scandinavica. 1990;69(4):301–6. 10.3109/00016349009036151 2244461

[38] Weitzman RE, Leake RD, Rubin RT, Fisher DA. The effect of nursing on neurohypophyseal hormone and prolactin secretion in human subjects. The Journal of Clinical Endocrinology Metabolism. 1980;51(4):836–9. 10.1210/jcem-51-4-836 7419669

[39] Velandia M. Parent-infant skin-to-skin contact studies: Parent-infant interaction and oxytocin levels during skin-to-skin contact after Cesarean section and mother-infant skin-to-skin contact as treatment for breastfeeding problems. Doctoral thesis.Karolinska Institute, Dept of Women's and Children's Health; 2012, Stockholm Sweden. https://openarchive.ki.se/xmlui/bitstream/handle/10616/40879/Thesis_Marianne_Velandia.pdf?sequence = 1&isAllowed = y

[40] Yokoyama Y, Ueda T, Irahara M, Aono T. Releases of oxytocin and prolactin during breast massage and suckling in puerperal women. European journal of obstetrics, gynecology, and reproductive biology. 1994;53(1):17–20. Epub 1994/01/01. 10.1016/0028-2243(94)90131-7 . 8187915

[41] Zinaman MJ, Queenan JT, Labbok MH, Albertson B, Hughes V. Acute Prolactin and Oxytocin Responses and Milk Yield to Infant Suckling and Artificial Methods of Expression in Lactating Women. Pediatrics. 1992;89(3):437 1741218

[42] Yuksel B, Ital I, Balaban O, Kocak E, Seven A, Kucur SK. Immediate breastfeeding and skin-to-skin contact during cesarean section decreases maternal oxidative stress, a prospective randomized case-controlled study. The Journal of Maternal-Fetal Neonatal Medicine. 2016;29(16):2691–6. 10.3109/14767058.2015.1101447 26415029

[43] Handlin L, Jonas W, Petersson M, Ejdebäck M, Ransjö-Arvidson A-B, Nissen E, et al Effects of sucking and skin-to-skin contact on maternal ACTH and cortisol levels during the second day postpartum—influence of epidural analgesia and oxytocin in the perinatal period. Breastfeeding medicine. 2009;4(4):207–20. 10.1089/bfm.2009.0001 19731998

[44] Handlin L, Jonas W, Ransjö-Arvidson A-B, Petersson M, Uvnäs-Moberg K, Nissen E. Influence of common birth interventions on maternal blood pressure patterns during breastfeeding 2 days after birth. Breastfeeding Medicine. 2012;7(2):93–9. 10.1089/bfm.2010.0099 22313391

[45] Jonas W, Nissen E, Ransjö-Arvidson AB, Wiklund I, Henriksson P, Uvnäs-Moberg K. Short-and long-term decrease of blood pressure in women during breastfeeding. Breastfeeding Medicine. 2008;3(2):103–9. 10.1089/bfm.2007.0031 18563998

[46] Widström A-M, Matthiesen A-S, Winberg J, Uvnäs-Moberg K. Maternal somatostatin levels and their correlation with infant birth weight. Early human development. 1989;20(3–4):165–74. 10.1016/0378-3782(89)90002-9 2575027

[47] Widström A-M, Werner S, Matthiesen AS, Svensson K, Uvnäs-Moberg K. Somatostatin levels in plasma in nonsmoking and smoking breast‐feeding women. Acta Pædiatrica. 1991;80(1):13–21. 10.1111/j.1651-2227.1991.tb11723.x 1674185

[48] Szeto A, McCabe P, Nation D, Tabak B, Rossetti M, McCullough M, et al Evaluation of enzyme immunoassay and radioimmunoassay methods for the measurement of plasma oxytocin. Psychosomatic medicine. 2011;73(5):393 10.1097/PSY.0b013e31821df0c2 21636661 PMC3118424

[49] Uvnäs-Moberg K, Handlin L, Kendall-Tackett K, Petersson M. Oxytocin is a principal hormone that exerts part of its effects by active fragments. Medical Hypotheses. 2019;(109394). 10.1016/j.mehy.2019.109394 31525634

[50] White‐Traut R, Watanabe K, Pournajafi‐Nazarloo H, Schwertz D, Bell A, Carter CS. Detection of salivary oxytocin levels in lactating women. Developmental Psychobiology: The Journal of the International Society for Developmental Psychobiology. 2009;51(4):367–73. 10.1002/dev.20376 PMC2767206 19365797

[51] Jonas, Nissen E, Ransjö-Arvidson A, Matthiesen A, Uvnäs-Moberg K. Influence of oxytocin or epidural analgesia on personality profile in breastfeeding women: a comparative study. Archives of women's mental health. 2008;11(5–6):335–45. 10.1007/s00737-008-0027-4 18726143

[52] Af Klinteberg B, Schalling D, Magnusson D. Self reported assessment of personality traits. Data from the KSP inventory on a representative sample of normal male and female subjects within a developmental project. In Reports from the project individual development and adjustment. Department of Psychology, University of Stockholm, 1986.

[53] Gustavsson P. Stability and validity of self-reported personality traits: Department of clinical neuroscience and institute for environmental medicine, Karolinska Institutet, Stockholm 1997.

[54] Heinrichs M, Neumann I, Ehlert U. Lactation and stress: protective effects of breast-feeding in humans. Stress. 2002;5(3):195–203. 10.1080/1025389021000010530 12186682

[55] Uvnäs-Moberg K. The gastrointestinal tract in growth and reproduction. Scientific American. 1989;261(1):78–83. 10.1038/scientificamerican0789-78 2568686

[56] Uvnäs Moberg K, Prime DK. Oxytocin effects in mothers and infants during breastfeeding. Infant. 2013;9(6):201–6.

[57] Prime DK, Geddes DT, Hepworth AR, Trengove NJ, Hartmann PEJBM. Comparison of the patterns of milk ejection during repeated breast expression sessions in women. 2011;6(4):183–90. 10.1089/bfm.2011.0014 21770734

[58] Strathearn L, Fonagy P, Amico J, Montague PR. Adult attachment predicts maternal brain and oxytocin response to infant cues. Neuropsychopharmacology. 2009;34(13):2655 10.1038/npp.2009.103 19710635 PMC3041266

[59] Keverne B, Kendrick K. Maternal behaviour in sheep and its neuroendocrine regulation. Acta Paediatrica. 1994;83:47–56. 10.1111/j.1651-2227.1994.tb13265.x 7981474

[60] Mezzacappa ES, Kelsey RM, Katkin ES. Breast feeding, bottle feeding, and maternal autonomic responses to stress. Journal of psychosomatic research. 2005;58(4):351–65. 10.1016/j.jpsychores.2004.11.004 15992571

[61] Rahm VA, Hallgren A, Högberg H, Hurtig I, Odlind V. Plasma oxytocin levels in women during labor with or without epidural analgesia: a prospective study. Acta Obstetricia et Gynecologica Scandinavica. 2002;81(11):1033–9. 10.1034/j.1600-0412.2002.811107.x 12421171

[62] Kennell JH, Trause MA, Klaus MH, editors. Evidence for a sensitive period in the human mother. Ciba Found Symp; 1975: Wiley Online Library. 10.1002/9780470720158.ch6 1045988

[63] Victora CG, Bahl R, Barros AJ, França GV, Horton S, Krasevec J, et al Breastfeeding in the 21st century: epidemiology, mechanisms, and lifelong effect. 2016;387(10017):475–90. 10.1016/S0140-6736(15)01024-7 26869575

[64] Bonifacino E, Schwartz EB, Jun H, Wessel CB, Corbelli JA. Effect of lactation on maternal hypertension: A systematic review. Breastfeeding Medicine. 2018;13(9):578–88. 10.1089/bfm.2018.0108 30299974

[65] Jacobson LT, Hade EM, Collins TC, Margolis KL, Waring ME, Van Horn LV, et al Breastfeeding history and risk of stroke among parous postmenopausal women in the Women's Health Initiative. Journal of the American Heart Association. 2018;7(17):e008739 10.1161/JAHA.118.008739 30371157 PMC6201437

[66] Rajaei S, Rigdon J, Crowe S, Tremmel J, Tsai S, Assimes TL. Breastfeeding duration and the risk of coronary artery disease. Journal of Women's Health. 2019;28(1):30–6. 10.1089/jwh.2018.6970 30523760 PMC6422010

[67] Victora C. Breastfeeding as a biological dialogue. Arch Argent Pediatr. 2017;115(5):413–4. Epub 2017/09/13. 10.5546/aap.2017.eng.413 . 28895686

[68] World Health Organisation. Ten steps to successful breastfeeding https://www.who.int/activities/promoting-baby-friendly-hospitals/ten-steps-to-successful-breastfeeding2020 [cited 2020 27 April].Full-text articles included in the study(n = 29)Included

