# Supplementary material for: Maternal plasma levels of oxytocin during breastfeeding—A systematic review
Source: PLoS One. 2020 Aug 5;15(8):e0235806. doi: 10.1371/journal.pone.0235806 (PMC7406087; doi:10.1371/journal.pone.0235806)
Supplement: S1 File — (DOCX) [file pone.0235806.s002.docx]

S1 File

Search strategy, 26^th^ September 2017

This search string was devised by combining the search

Oxytocin

Levels

Blood

Pregnancy/labour/birth/interventions/postpartum/fetus/infant/breastfeeding etc

(see Search A below)

with AND terms for

Breastfeeding

These searches were combined into Covidence (453) and 40 duplicates automatically removed, giving 403 for screening

PUBMED 305 results

((((((( "Blood/analysis"[Mesh] OR "Blood/blood"[Mesh] OR "Blood/chemistry"[Mesh] OR "Blood/diagnosis"[Mesh] OR "Blood/drug effects"[Mesh] OR "Blood/pathology"[Mesh] OR "Blood/physiopathology"[Mesh] OR "Blood/statistics and numerical data"[Mesh] OR "Blood/toxicity"[Mesh] ))) OR ("Diagnostic Techniques and Procedures/blood"[Mesh])) OR "Biological Assay"[Mesh:NoExp]) OR plasma[tiab]) OR "Plasma"[Mesh]) OR circulat*[tiab]) OR blood*[tiab])) AND ((((oxytocin[tiab] OR oxytocin[nm] OR "Oxytocin"[Mesh] OR Pitocin[tiab] OR syntocin*[tiab])) AND (((((Concentration[tiab] OR Level[tiab] OR Amount[tiab] OR Quantity[tiab] OR Sum[tiab] OR Total[tiab] OR RIA[tiab] OR *assay[tiab] OR Measure*[tiab])))))))) AND (("Pregnancy"[Mesh] OR "Parturition"[Mesh] OR (("Postpartum Period"[Mesh] OR "Obstetric Labor Complications"[Mesh] OR "Delivery, Obstetric"[Mesh] OR "Labor, Obstetric"[Mesh] OR "Analgesia, Epidural"[Mesh] OR "Labor, Induced"[Mesh] OR "Fetus"[Mesh] OR "Obstetric Labor Complications"[Mesh] OR "Milk, Human"[Mesh] OR "Breast Feeding"[Mesh] OR "Lactation"[Mesh] OR "Parturition"[Mesh] OR "Delivery, Obstetric"[Mesh] OR intrapartum[Title/Abstract]) OR newborn[Title/Abstract] OR neonat*[Title/Abstract] OR "Infant, Newborn"[Mesh] OR labor[Title/Abstract] OR labour[Title/Abstract] OR caesarean[Title/Abstract] OR cesarean[Title/Abstract] OR birth [Title/Abstract] or obstetric*[Title/Abstract] OR postnatal[Title/Abstract] OR post-natal[Title/Abstract] OR postpartum[Title/Abstract] OR epidural[Title/Abstract] OR breastfeed*[Title/Abstract] OR lactati*[Title/Abstract])))) AND (("Milk, Human"[Mesh] OR "Breast Feeding"[Mesh] OR "Lactation"[Mesh] OR breastfeed*[Title/Abstract] OR lactati*[Title/Abstract]))))

SCOPUS 91 results

## ( ( TITLE-ABS-KEY ( oxytocin  OR  syntocin*  OR  pitocin ) )  AND  ( ( TITLE-ABS-KEY ( level*  OR  assay  OR  ria  OR  radioimmunoassay  OR  sum  OR  total  OR  measure*  OR  concentrat*  OR  amount  OR  quantity ) ) )  AND  ( TITLE-ABS-KEY ( blood  W/3  level*  OR  plasma  W/3  level*  OR  circulat*  W/3  level*  OR  plasma ) )  AND  ( TITLE-ABS-KEY ( postpart*  OR  postnatal*  OR  puerperium  OR  intrapart*  OR  perinat*  OR  labor*  OR  labour*  OR  birth*  OR  childbirth*  OR  parturit*  OR  pregnancy  OR  cesarean*  OR  caesarean*  OR  neonat*  OR  infant*  OR  fetus  OR  fetal  OR  delivery  OR  epidural  OR  breastfeed*  OR  obstetric*  OR  umbilic*  OR  ( induc*  W/3  labo*r )  OR  ( augment*  W/3  labo*r )  OR  forceps  OR  amniotom*  OR  "artificial rupture of membranes"  OR  ( synthetic  W/3  oxytoci* )  OR  ( breast*  W/3  feed* )  OR  ( breast*  W/3  lactat* )  OR  ( breast*  W/3  milk* )  OR  ( lactat*  W/3  milk* )  OR  breastfeed* ) ) )  AND  ( ( TITLE-ABS-KEY ( ( breast*  W/3  feed* )  OR  ( breast*  W/3  lactat* )  OR  ( breast*  W/3  milk* )  OR  ( lactat*  W/3  milk* )  OR  ( human*  W/3  milk* ) ) )  OR  ( TITLE-ABS-KEY ( breastfeed* ) ) )

CINAHL 7 results

((TI ( pitocin OR syntocin* OR oxytocin) OR AB ( pitocin OR syntocin* OR oxytocin) OR MH oxytocin) AND (TI ( level OR concentrat* OR amount OR sum OR total OR measure OR *assay OR quantity OR RIA) OR AB (level OR concentrat* OR amount OR sum OR total OR measure OR *assay OR quantity OR RIA) OR MH (("Biological Assay") OR ("Radioimmunoprecipitation Assay") OR ("Enzyme-Linked Immunosorbent Assay"))) AND (TI ( Plasma OR blood* OR circulat*) OR AB ( Plasma OR blood* OR circulat*)) AND ((MH childbirth) OR (MH "Postnatal Period+") OR (MH "Pregnancy+") OR TI labo*r OR AB labo*r OR TI post*natal OR AB post*natal OR TI post*partum OR AB post*partum OR TI c*esarean OR AB c*esarean OR TI newborn OR AB newborn OR TI neonat* OR AB neonat* OR TI infant* OR AB infant* )) AND ((MH "Breast Feeding+") OR TI breast*feed* AND AB breast*feed* OR AB lactati* OR TI lactati* OR TI breast N2 milk OR AB breast N2 milk OR TI human N2 milk OR AB human N2 milk)

PSYCINFO 50 results

((title: (breastfeed*) OR title: (lactati*)) OR (abstract: (breastfeed*) OR abstract: (lactati*)) OR (Keywords: (breastfeed*) OR Keywords: (lactati*))) AND ((((title: (postpart*)) OR (title: (postnatal*)) OR (title: (puerperium)) OR (title: (intrapart*)) OR (title: (perinat*)) OR (title: (labor*)) OR (title: (labour*)) OR (title: (birth*)) OR (title: (childbirth*)) OR (title: (parturit*)) OR (title: (pregnancy)) OR (title: (cesarean*)) OR (title: (caesarean*)) OR (title: (neonat*)) OR (title: (infant*)) OR (title: (fetus)) OR (title: (fetal)) OR (title: (delivery)) OR (title: (epidural)) OR (title: (breastfeed*)) OR (title: (obstetric*)) OR (title: (umbilic*)) OR (title: (forceps)) OR (title: (amniotom*)) OR (title: ("artificial rupture of membranes")) OR (title: (breastfeed*))) OR ((abstract: (postpart*)) OR (abstract: (postnatal*)) OR (abstract: (puerperium)) OR (abstract: (intrapart*)) OR (abstract: (perinat*)) OR (abstract: (labor*)) OR (abstract: (labour*)) OR (abstract: (birth*)) OR (abstract: (childbirth*)) OR (abstract: (parturit*)) OR (abstract: (pregnancy)) OR (abstract: (cesarean*)) OR (abstract: (caesarean*)) OR (abstract: (neonat*)) OR (abstract: (infant*)) OR (abstract: (fetus)) OR (abstract: (fetal)) OR (abstract: (delivery)) OR (abstract: (epidural)) OR (abstract: (breastfeed*)) OR (abstract: (obstetric*)) OR (abstract: (umbilic*)) OR (abstract: (forceps)) OR (abstract: (amniotom*)) OR (abstract: ("artificial rupture of membranes")) OR (abstract: (breastfeed*))) OR ((Keywords: (postpart*)) OR (Keywords: (postnatal*)) OR (Keywords: (puerperium)) OR (Keywords: (intrapart*)) OR (Keywords: (perinat*)) OR (Keywords: (labor*)) OR (Keywords: (labour*)) OR (Keywords: (birth*)) OR (Keywords: (childbirth*)) OR (Keywords: (parturit*)) OR (Keywords: (pregnancy)) OR (Keywords: (cesarean*)) OR (Keywords: (caesarean*)) OR (Keywords: (neonat*)) OR (Keywords: (infant*)) OR (Keywords: (fetus)) OR (Keywords: (fetal)) OR (Keywords: (delivery)) OR (Keywords: (epidural)) OR (Keywords: (breastfeed*)) OR (Keywords: (obstetric*)) OR (Keywords: (umbilic*)) OR (Keywords: (forceps)) OR (Keywords: (amniotom*)) OR (Keywords: ("artificial rupture of membranes")) OR (Keywords: (breastfeed*)))) AND (((title: (blood)) OR (title: (plasma)) OR (title: (circulat*))) OR ((abstract: (blood)) OR (abstract: (plasma)) OR (abstract: (circulat*))) OR ((Keywords: (blood)) OR (Keywords: (plasma)) OR (Keywords: (circulat*)))) AND (((title: (: level*)) OR (title: (assay)) OR (title: (ria)) OR (title: (radioimmunoassay)) OR (title: (sum)) OR (title: (total)) OR (title: (measure*)) OR (title: (concentrat*)) OR (title: (amount)) OR (title: (quantit))) OR ((abstract: (: level*)) OR (abstract: (assay)) OR (abstract: (ria)) OR (abstract: (radioimmunoassay)) OR (abstract: (sum)) OR (abstract: (total)) OR (abstract: (measure*)) OR (abstract: (concentrat*)) OR (abstract: (amount)) OR (abstract: (quantit))) OR ((Keywords: (: level*)) OR (Keywords: (assay)) OR (Keywords: (ria)) OR (Keywords: (radioimmunoassay)) OR (Keywords: (sum)) OR (Keywords: (total)) OR (Keywords: (measure*)) OR (Keywords: (concentrat*)) OR (Keywords: (amount)) OR (Keywords: (quantit)))) AND (((title: (oxytocin)) OR (title: (pitocin)) OR (title: (syntocin*))) OR ((abstract: (oxytocin)) OR (abstract: (pitocin)) OR (abstract: (syntocin*))) OR ((Keywords: (oxytocin)) OR (Keywords: (pitocin)) OR (Keywords: (syntocin*)))))
